# Adaptation to climate change in the Ontario public health sector

**DOI:** 10.1186/1471-2458-12-452

**Published:** 2012-06-19

**Authors:** Jaclyn A Paterson, James D Ford, Lea Berrang Ford, Alexandra Lesnikowski, Peter Berry, Jim Henderson, Jody Heymann

**Affiliations:** 1 Department of Geography, McGill University, Burnside Hall, 805 Sherbrooke Street West, Montreal, QC, H3A 2 K6, Canada; 2 Climate Change and Health Office, Health Canada, 269 Laurier Avenue West, Ottawa, ON, K1A 0 K9, Canada; 3 Life Sciences Library, McIntyre Medical Building, McGill University, 3655 Promenade Sir William Osler, Montreal, QC, H3G 1Y6, Canada; 4 Institute for Health and Social Policy, Meredith, Charles, House, 1130 Pine Avenue West, Montreal, QC, H3A 1A3, Canada

**Keywords:** Climate change, Health, Adaptation, Ontario, Canada

## Abstract

**Background:**

Climate change is among the major challenges for health this century, and adaptation to manage adverse health outcomes will be unavoidable. The risks in Ontario – Canada’s most populous province – include increasing temperatures, more frequent and intense extreme weather events, and alterations to precipitation regimes. Socio-economic-demographic patterns could magnify the implications climate change has for Ontario, including the presence of rapidly growing vulnerable populations, exacerbation of warming trends by heat-islands in large urban areas, and connectedness to global transportation networks. This study examines climate change adaptation in the public health sector in Ontario using information from interviews with government officials.

**Methods:**

Fifty-three semi-structured interviews were conducted, four with provincial and federal health officials and 49 with actors in public health and health relevant sectors at the municipal level. We identify adaptation efforts, barriers and opportunities for current and future intervention.

**Results:**

Results indicate recognition that climate change will affect the health of Ontarians. Health officials are concerned about how a changing climate could exacerbate existing health issues or create new health burdens, specifically extreme heat (71%), severe weather (68%) and poor air-quality (57%). Adaptation is currently taking the form of mainstreaming climate change into existing public health programs. While adaptive progress has relied on local leadership, federal support, political will, and inter-agency efforts, a lack of resources constrains the sustainability of long-term adaptation programs and the acquisition of data necessary to support effective policies.

**Conclusions:**

This study provides a snapshot of climate change adaptation and needs in the public health sector in Ontario. Public health departments will need to capitalize on opportunities to integrate climate change into policies and programs, while higher levels of government must improve efforts to support local adaptation and provide the capacity through which local adaptation can succeed.

## Background

Climate change has been identified as one of the major challenges for health this century [1]. The risks in Canada include increasing temperatures, more frequent and intense extreme weather, and alterations to precipitation regimes [2]. While Arctic impacts will be most pronounced, southern provinces, including Ontario, will also experience changing weather patterns. Projections indicate that:

· Changing temperature and precipitation regimes will increase the probability and severity of extreme events including heatwaves, storms, floods, drought, and wildfires with implications for asthma, chronic respiratory disease, cardiovascular disease, and water quality and contamination [3-5].

· Warmer, wetter summers will affect the distribution and survival of pathogens and some disease vectors such as mosquitoes and ticks, [6], with implications for Lyme disease [7,8], West Nile virus [9], and waterborne diseases [10].

· Rising temperatures will increase the incidence of temperature dependant food-borne diseases including *Salmonella* and toxin producing organisms such as *Staphylococcus aureus* and *Clostridium botulinum*[5,6,11].

· There will also be indirect pathways through which climate change will affect health, although these remain poorly researched [12]. Changing frequency and magnitude of extreme weather events, for example, could affect socio-cultural well-being and mental health [3].

In light of these risks, and the inevitability of some degree of future change, public health systems in Canada and beyond will have to adapt to manage climate-related risks [3,13-16]. Interventions will be needed to reduce the burden of climate-sensitive health outcomes [1,17]. A number of trends could magnify the implications climate change has for Canada’s most populous province, including the presence of rapidly growing vulnerable populations, exacerbation of warming trends by heat-islands in large urban areas and connectedness to global transportation networks (and hence disease importation) [18,19]. Eighty five percent of Ontario’s 13.3 million residents live in urban centers, and in the densely populated southeastern region characterized by industrialized zones and sprawling suburbs [18]. The magnitude of the adaptation challenge is emphasized in the Report of the Expert Panel on Climate Change Adaptation [20] which contributed to the establishment and recent release of the province’s adaptation action plan in 2011 [21].

While previous research has begun to examine national level adaptation, few studies have examined the response of local policy makers [22]. This lack of information is part of a broader ‘adaptation deficit’ identified by Burton (2006), and constrains efforts to understand the magnitude of the challenge posed by climate change and limits the ability to effectively plan for adaptation [2,3,23-25]. Indeed, in the climate and health scholarship, there are few studies that have documented the perspectives of practitioners on adaptation, with adaptation typically discussed in an abstract sense [26]. In Ontario, health hazard management includes a requirement for boards of health to increase public awareness of health risk factors associated with climate change [27]. As well, regional and local municipal officials in emergency planning, water and waste management, environment and conservation and municipal planning also play important roles in managing health risks of climate change.

This paper contributes towards addressing this deficit in research on local responses in general and in Canada specifically, by developing a baseline understanding of the status of adaptation in the Ontario health sector. The research uses in-depth interviews with actors in public health and regional and local officials to: i) establish an understanding of the experiences, perceptions, and processes of adaptation to climate change in the health sector; ii) identify responses being considered or implemented; and iii) identify and characterize opportunities and barriers to adaptation. The focus is on public health managers implementing policy at the provincial and regional levels which reflects the structure of the Canadian health care system, with health powers shared across jurisdictions but primarily provincial. In Ontario, responsibilities are outlined in the Health Protection and Promotion Act (1990) which provides the legislative mandate for 36 regional boards of health and associated standards for the provision of mandatory public health programs and services with which every board of health (regional) is required to comply [28]. In the area of climate change, both Health Canada and the Public Health Agency of Canada (PHAC) play a role at the federal level. Municipalities, provinces/territories and the federal government share roles significant to climate change adaptation including water quality, food safety, infectious disease control, air pollution and heat waves, natural hazards and general health promotion and disease prevention [29].

## Methods

**Figure 1:**
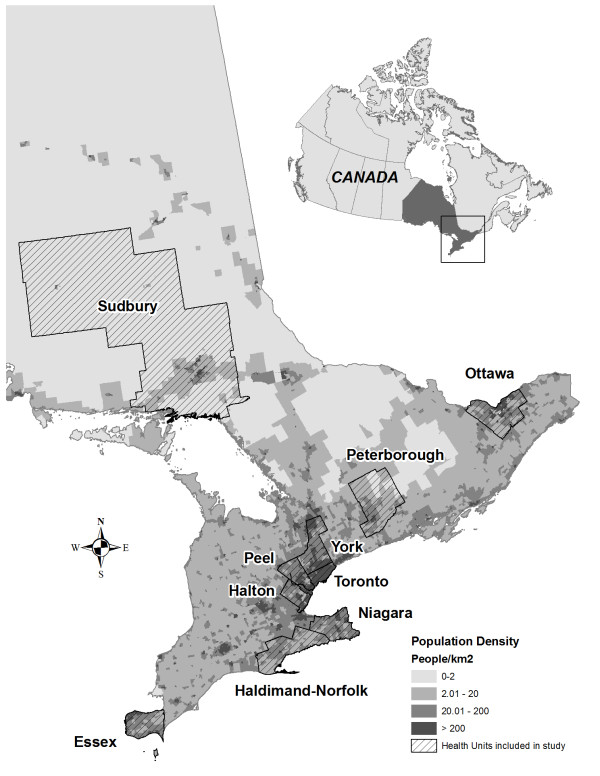
Population density map of Ontario illustrating public health jurisdictions included in this study.

Fifty-three semi-structured interviews were conducted in 2010, four with provincial and federal health officials and 49 with actors in public health and health relevant sectors at the municipal level. Interview recruitment was designed to include respondents from north, central and southern Ontario. Most respondents were from jurisdictions of central and southern Ontario as these regions are more highly populated and are characterized by an urban rural mix which is indicative of much of the province. Refer to Additional file 1 for population and geographic characteristics of regions included in this study. Purposive sampling of government officials in public health (federal, provincial, regional), emergency management, planning, water, and environment/conservation at the regional or municipal level was carried out. Climate change and health relevant responsibilities in each sector are found in Additional file 2. Departments selected were involved in adaptation initiative(s) addressing one or more health vulnerabilities of climate change. The municipality needed to have an adaptation strategy planned or implemented and the public health department needed to be involved in the adaptation process. Interviewees were senior level staff, researchers and policy analysts. See Additional files 3 and/or 4 for criteria for selection of regional health jurisdictions and interview participants. Ten health jurisdictions were included covering 66 of 444 municipalities in Ontario. Refer to Figure 1 to view a population density map of Ontario indicating the public health jurisdictions included in this study and Additional file 1 specifying the numbers of interviewees per health jurisdiction. Municipal interviews were complemented by interviews with federal (n = 2) and Ontario provincial (n = 2) officials in the public health sector. Federal public health departments or agencies that are active in researching ways to support climate change adaptation in Canada were selected to facilitate characterization of national efforts and their influence on local adaptation now and in the future. Ontario provincial public health ministries’ were targeted to assess the priority given to climate change when developing provincial public health policies, standards and guidance materials. Representative(s) from the Ministry of Health and Long-term Care were unavailable for interviews; as such, pertinent information was obtained from the Ministry's website.

Interviews were conducted in person or by telephone, were recorded, and lasted 45–90 minutes. To characterize adaptation experiences, a typology adapted from Smit et al. (1999, 2000) and Berrang-Ford et al. (2011) were utilized as a guide to address 7 main adaptation themes: (1) General information, which aimed to identify area(s) of responsibility including number of staff, population base, and governance structure; (2) climate change priority and health risks of concern; (3) characterization of adaptation actions, including motivation, process, resources, stakeholders, targeted population, monitoring, challenges and drivers; (4) adequacy of top-down support to facilitate local climate change adaptation; (5) perceptions of agency capacity at the local level to adapt to health risks of climate change; (6) roles and responsibilities for climate change adaptation; and (7) suggestions on opportunities for improved or more effective adaptation capacity [24,30,31]. Questions were posed to address each theme (see Additional file 5) and a codebook of definitions was used to categorize interview responses (see Additional file 6). The codebook allowed descriptive statistics to be calculated whereby the number of responses per theme and question were tabulated.

Interviews were complimented by a literature review using relevant key words in the search engine ISI Web of Knowledge (including Medline) and a scan of government websites considered relevant to climate change and public health. The literature review was used to: (a) enrich knowledge on public health adaptation in Canada and (b) to inform the questionnaire; specifically, to expand upon existing adaptation typologies [24,30,31], to identify relevant questions per typology theme and to populate the codebook with definitions prior to conducting interviews.

Approval to conduct this research was obtained by the Research Ethics Board Office at McGill University prior to recruitment of interviewees and data collection.

## Results

### Ontario public health officials are most concerned about extreme weather and air quality

Regional health officials (n = 28) identified extreme heat (n = 20, 71%), storms and floods (n = 19, 68%) and poor air quality (n = 16, 57%) as the most pressing climate change health risks. Heat and air quality emerged as separate concerns noting that more frequent and intense hot days will increase smog with negative implications for cardiovascular and respiratory health especially for vulnerable groups. Associated with these risks were concerns about ageing populations, urban sprawl and the heat island effect.

"With climate change, heat could be the big threat given our ageing population. I am concerned about knowing where vulnerable groups are and if they have social support. I am unsure if the vulnerable groups will be taken care of and of the adequacy of the current system to ensure appropriate responses to these peoples’ needs to be assessed (Municipal public health official)"

Concern of direct impacts of storms and floods included increased injuries due to slip and fall accidents in icy conditions, and injuries or deaths from extreme events. Indirect health impacts identified included: respiratory problems from poor indoor air quality and psychological effects from damage to homes and buildings after a flood (e.g. City of Windsor, 2010 flood); water-borne illnesses through direct contact with contaminated recreational water; and social and economical burdens associated with property damage or critical infrastructure failure. Respondents noted isolated flood events in some areas that were developed in flood plains prior to implementation of land development regulations. Other concerns included potential costs of repairing damaged buildings and roads, storm-water overflows in cities with combined sewer systems, and private water system contamination. Officials stated that the built environment is often based on out-dated codes and standards that do not integrate climate change considerations. Most regional officials stated that while emergency management measures are robust to manage climate change impacts, many are unsure if infrastructure will withstand extreme events (e.g. 2005 flood in Toronto, 2004 flood in Peterborough). In all southern jurisdictions, air pollution, including managing pollutant sources outside of their jurisdiction is of great concern.

Other health vulnerabilities identified were vector-borne diseases (n = 8, 29%), food contamination (n = 5, 18%), and water contamination (n = 5, 18%). How best to manage climate change ‘creeping effects’ on food and water safety, and vector borne diseases was an issue for some.

"Climate change happens on two fronts – the sudden acute events, like extreme weather events and so on, and then, there is the slow creeping incremental change. We are probably better suited because we have done the emergency management plans and thought through some of that stuff. We are probably equipped to handle the acute stuff. But the creeping effects, we’re not really sure. We are identifying all of the impacts and so I’m personally not so comfortable on that level (Municipal public health official)"

Concerns about climate change were often linked with past occurrences of heat or air related mortality, isolated events or trends in weather patterns associated with more intense drought, wind storms (e.g. tornadoes) or floods.

### Climate change is not addressed as a stand-alone issue, but adaptation is apparent

In Ontario, climate change is an implicit or mainstreamed consideration in public health initiatives where evidence of climate-related risks is available. Adaptation was described as a newly evolving process, as more information becomes available, respondents noted that mainstreaming will become more prevalent.

"We are at the stage of what do we need to be concerned about, how do we start to integrate into our long-term planning program; we recognize it is not where it needs to be, but it's an evolving process (Municipal public health official)"

Public health officials identified activities (n = 39) relevant to managing climate change health risks, most of which were provincially mandated programs originally established without a climate change lens. Among public health department program initiatives, 10 (26%) addressed extreme temperatures and 7 (18%) air quality. Others included food (n = 5, 13%), vector-borne diseases (n = 5, 13%) water (n = 4, 10%), public health emergency management (n = 2, 5%) and prevention of ultraviolet radiation (n = 1, 3%) programs. The remaining 5 were explicit climate change adaptation initiatives or plans implemented in the health department (n = 2, 5%) or externally (n = 3, 8%). These included a planned climate change and health impact assessment (Halton), an environmental health survey embedding public risk perceptions of climate change (York), an Ontario climate change task force, a regional climate change adaptation strategy (Peel) and an adaptation consortium (Sudbury).

Health department respondents described strategies addressing climate change health vulnerabilities. Extreme heat programs use alert and warning systems, planning and response initiatives, promotional messaging to the public and response guidelines on preventing illness or death and monitoring weather to inform heat alerts. To manage extreme temperatures, the City of Ottawa uses a multi-stakeholder extreme weather prevention and response strategy to maximize capacity for public protection, especially for those most vulnerable (e.g. elderly, homeless). Peel and Ottawa plan to use or have implemented syndromic surveillance of heat related hospital visits during extreme heat alerts to monitor burden of illness in hot weather. The Windsor-Essex Public Health Unit with the City of Windsor implemented a heat alert and response system and conducted a heat health vulnerability assessment to identify vulnerable groups, thresholds for issuing heat alerts and indicators of effective responses. Results from the City of Windsor’s heat alert and response system (HARS) pilot and Toronto Public Health’s efforts in evaluating effectiveness of heat messaging will contribute to a national HARS best practices guidebook developed by Health Canada.

Air quality management includes monitoring smog, issuing alerts and public education on illness prevention and energy reduction on smog days; burden of illness studies contribute to informing air quality policies and anti-idling bylaws. Food safety includes surveillance of food premises, public disclosure, warnings and recalls, education on safe food handling and food preparation, locally grown food and roof-top gardens. Greening initiatives as part of city plans have multiple benefits of filtering air-pollutants, promoting locally grown food, reducing the urban heat island effect, and storm-water management. Toronto green roofs, for example offer multiple benefits. The ‘Shade Policy’ was endorsed by Toronto city council, requiring provision of shade in city owned and operated outdoor venues for primary prevention of skin cancer and associated health burdens. The Greater Toronto Area (GTA) health units (Peel, Halton, York, Toronto) participate in the Clean Air and Climate Change Summit, where municipalities gather to exchange information and implement the 20/20 Way to Clean Air; an adaptation relevant public promotional campaign which encourages the public to reduce energy use at home and on the road by 20% (improve local air quality). York Region integrates climate change into considerations on a variety of policies and plans. In particular, climate change has been incorporated into comments on official plans, transportation plans, public health programs and environmental assessments. In Ottawa and Halton, processes are in place for land development policy to be informed by air quality monitoring, modeling and research conducted by the regional Health Units. In Halton, these efforts are part of an extensive Air Quality Program that includes educational climate change and air quality initiatives (e.g. Tools for Schools). Some health units (Peel, Halton, York, Toronto, Ottawa) noted their involvement in piloting the National Air Quality Health Index [32], a health based indicator of air quality combined with targeted messages to vulnerable groups on how to avoid smog related illnesses. Involvement in these pilots includes monitoring behavioral changes of citizens which will contribute to improvements in effective preventative messaging to the public.

Other adaptation examples addressed extreme weather events (storms and floods), safe water and vector-borne diseases. Emergency management uses risk assessments and stakeholder networking and response plan development. Risks are assessed using Hazard Impact Risk Assessments in the public health department and at the community level. The City of Greater Sudbury participated in a community climate change vulnerability assessment which informed adaptive capacity gaps and needs in health and other sectors. Subsequent initiatives included the newly established Sudbury Adaptation Consortium, a community based adaptation forum through which partners discuss adaptation needs. One project involves mapping vulnerable subgroups and critical infrastructure in relation to high risk flood and storm zones. Water safety management includes monitoring recreational water quality, overseeing the regular testing of private systems, and issuing beach closure and boiled water advisories. Niagara Public Health has adapted safe water and vector borne disease management in light of risks associated with changes in weather patterns. The Niagara Region health department is modeling water quality using predictive real time water monitoring stations and has installed an aerial surveillance system to monitor disease vectors in a changing climate. Health departments implement programs to manage vector-borne diseases, notably West Nile Virus, Eastern Equine Encephalitis, Lyme disease and imported disease cases like malaria. Niagara’s vector surveillance project will contribute to infectious disease surveillance and monitoring best practices for Canadian health departments (Public Health Agency of Canada). Peel Public Health implemented a West Nile Virus rapid risk factor surveillance system which includes a seasonal questionnaire distributed to residents.

### Key adaptation enablers include political will, inter-agency coordination and local leadership

Adaptation efforts rely on local political will and federal support, multidisciplinary partnerships and local leaders. Local government and senior management that support climate change adaptation were described as enablers, providing support and legitimacy for the adaptation implementation:

"[The municipal council] endorsement and support was important in gaining a community start [on climate change adaptation consortium], community awareness and perspective (Municipal public health official)"

Public health programs are provincially mandated but locally designed and operated, such that climate change initiatives rely on support from senior managers and/or the Medical Officer of Health. Provincial health standards that address climate change explicitly are minimal. The shade policy in Toronto, for example, was endorsed in part because it contributed to municipal climate change mitigation and adaptation goals. Federal government agencies have provided financial and/or informational support for implementing and sustaining local adaptation projects. Examples of federal programs include piloting heat alert and response systems and the Air Quality Health Index (AQHI), vector borne disease programming (surveillance and monitoring) and tools to address climate change health risks:

"We were already engaged and knew we needed to do more … The funding became available to do something through Public Health Agency of Canada. [This] funding was a big impetus [for the surveillance and monitoring system]. It gave us the ability to move forward on a much larger scale - we wouldn't have had the funding to do that kind of work otherwise (Municipal public health official)"

Adaptations in Ontario have capitalized on interdepartmental cooperation; municipal adaptation efforts include public health participation in discussions, brainstorming sessions and in developing adaptation plans. Climate change is integrated into official plans or strategies (e.g. York, Peel, Ottawa, and Toronto) toward which public health departments provide input and implement programs that contribute to municipality wide goals. Such efforts have benefited from existing inter-departmental frameworks. Others have made use of the emergency management planning committees to mainstream climate change. Local leaders enable adaptation via organizing and chairing meetings and obtaining informational and financial support to sustain projects and create mechanisms to make the case for climate change adaptation in the community.

"The climate change collaborative at York University has helped us. I have been offered assistance from all the municipalities around us. It is nice to have that support, to have their knowledge, the research expertise available and to be able to connect with their resources. I also hope to work with the Alliance for Resilient Cities and the Clean Air Partnership even more. These groups and interactions provide perspective that I didn't otherwise have (Municipal Planner)"

Expert judgment is considered important in the provision of knowledge for decision makers to prioritize adaptation activities, while conferences and workshops are helpful in identifying key concerns, sharing strategies and promoting and spreading awareness in the community.

"The vulnerability assessment workshops we organized [on extreme heat] were very helpful. The table top exercises were also very helpful. The continual workshops allowed issues to be raised over time. They were good venues because all stakeholders were there, and different and diverse groups were talking to one another (Municipal environment official)"

### Adaptation constraints include inadequate resources, a perceived lack of urgency and communication barriers

Respondents noted that limited resources, lack of urgency and communication barriers constrained adaptation. Sustained efforts are limited by short-term funding and human resources. Long-term funding needed for hiring personnel and obtaining necessary resources for adaptation is an inherent challenge when adaptation is not mandated provincially and where urgency to adapt may not be shared from one municipal council to another. Officials noted that short political terms hinder achievement of long-term goals required for adaptation. Advocates of adaptation are concerned about competing interests and more immediate issues taking precedence*.* Limited knowledge of how climate will change locally and the implications for health hinders urgency to implement plans and programs to prepare for the future. Currently, the resolution of climate change projection models is inadequate to inform adaptation at local scales. Knowledge of climate-related health outcomes, adaptation options and guidance are inadequate. Provincial health standards provide the framework for mainstreaming climate change into local programs; however, adaptation information per health vulnerability is lacking. Most notably, the ‘creeping’ nature of some climate change impacts brings uncertainty in knowing when water-borne or food borne outbreaks will occur, critical infrastructure will breakdown and/or when public health and emergency capacity will be inadequate to extreme events or disasters occurring in close succession. Health officials felt constrained by a lack of data and noted the need for more research. Furthermore, a lack of data or causal estimates of morbidity and mortality associated with environmental determinants or risks limit effective evidenced based programming. Public health officials noted inconsistencies in numbers of positive cases of West Nile Virus across communities, limiting predictability of human infection.

"We are supposed to anticipate health impacts from any potential health hazard and respond to it. Preparedness in terms of climate change for us means doing surveillance and putting plans in place in the event that something happens. We need to be confident with our plans. We need more environmental exposure data to guide our actions…. One of our hardest challenges deals with linking environment with health outcomes. Our work needs to be evidenced based; however, it’s difficult to associate respiratory outcomes with actual impact from poor air quality or linking water borne disease outbreaks with environment….. For climate change health risks that we don’t have evidence for yet, the funding won’t be there (Municipal public health officials)"

Effective messaging and communication of the health risks associated with climate change is a challenge. Inter-jurisdictional differences on use of triggers to inform alert and warning systems and conflicting messages to the public on extreme heat and smog days are significant issues. Difficulties exist in promoting public responses because climate change has not yet resonated in communities. Effective messaging is difficult in diverse communities and the public lacks a clear perception of personal risks and/or confuses concepts of adaptation and mitigation. Monitoring adequate responses of vulnerable groups is also challenging. Furthermore, health officials have voiced the need for standardized and evidenced based best practices for evaluating programs.

"There is no way of monitoring the success of our extreme weather program. We need an evaluation strategy - every year we question the validity of this. Nowadays, you have to have research-based evidence of effectiveness and climate related extreme events(Municipal public health official)"

Table 1 lists research gaps and needs pertaining to climate change adaptation in Ontario as indicated by public health respondents. Additional File 7 provides examples of additional quotes from officials interviewed in this study.

**Table 1:** Public health adaptation knowledge gaps in Ontario as indicated by public health respondents

**Field**	**Public Health Adaptation Knowledge Gaps**
Information, Science & Technology	Resolution of climate modelling to local scales
	Research on climate change science and health impacts locally
	Surveillance of environmental exposure and health impacts
	Guidance on mainstreaming climate change adaptation
	Tools to build awareness, convey messages effectively and stress urgency to act
	Cost-effective, win-win adaptation strategies that account for diversity and complexity
	Public health adaptation best practices & lessons learned; identified and widely accessible
	Research on relevant triggers to inform warning systems
Infrastructure	Assessments of resiliency of healthcare and emergency management infrastructure
	Assessments of adequacy of building code standards in light of climate change
	Research on required land development policies in light of climate change
	Studies on thresholds of water utilities infrastructure and transportation networks
Human Resources & Institutions	Cost-benefit analyses of adapting versus status quo
	Assessments of costs, direct & indirect, short & long-term health impacts
	Policies to ensure sustained funding of climate change research, adaptation programs & plans
	Tools to facilitate sustained community climate change knowledge sharing and planning
	Assessments of local capacity to prepare for health related impacts of climate change
	Mechanisms to evaluate effectiveness of programs in reducing health risks of climate change

### More top-down support is needed for adaptation

When asked to prioritize developments needed for adaptation, public health respondents indicated increased supporting roles by the provincial and federal government. Among 28 public health officials, 13 felt the Ontario public health standards were adequate in providing the framework to adapt to climate change; while 15 felt the standards could improve by including clearer guidelines for climate change adaptation. Many health officials (n = 19, 61%) felt they lacked information to comment on the adequacy of federal efforts to support local adaptation; while 6 (19%) believed federal efforts were adequate and 6 (19%) stated that they were not. Existing government support in the form of funding, adaptation toolkits and information on climate change planning was indicated as very helpful. Also, including ‘climate change’ in Ontario health standards has provided legitimacy to allocate funding to adaptation initiatives. Public health officials identified how higher levels of government could improve in supporting local adaptation. Of the 31 public health respondents, 14 stated the need for adaptation inventories, 10 mentioned increased funding for research and local adaptation efforts; 6 expressed the need for improved provincial environmental surveillance and monitoring systems, and improved guidance and protocols were mentioned by 4 respondents.

"We don’t have the resources to do trial and error of what works and what doesn’t. What we need is a one stop shop and easy at our fingertips best practices for making plans for these health hazard emergencies (Municipal public health official)"

Many noted the “Human Health in a Changing Climate” report produced by Health Canada [3] as an important first step, but that more specific and tangible guidance for local adaptation is needed; some officials who were aware of Health Canada’s initiatives to increase the resilience of communities to extreme heat events are looking forward to applying tools and best practices to existing heat programs. Many felt national leadership in sustaining the urgency to adapt would help to ensure continued effort at the municipal level, potentially facilitated by a national adaptation strategy. Health officials stated that more support needs to come from the Ontario Health Ministries, firstly, in the form of more emphasis on climate change in standards, but also including more research. It was noted that prioritized efforts at higher levels of government should include improving resolution of climate change models for increased applicability at more local scales. Provincial and federal officials noted that health units are key players and partners in adapting to climate change; they are well positioned to identify local issues and can capitalize on existing programs and resources to proactively address them. However, they acknowledge the limitations of local health departments and the need for continued efforts to ensure that public health departments in Canada are prepared for a changing.

"The province needs a more rigorous review on what the most likely impacts of climate change are that have implications on public health in Ontario in next 50 years and what needs to be done to respond to that and reduce those risks. ......I think providing evidence and best practices in the area of local climate change is certainly something we want to do here. (Provincial public health official)"

## Discussion and conclusion

This study provides a snapshot of climate change adaptation in the public health sector in Ontario and results will help inform future efforts to facilitate adaptation. Limitations of the study firstly include more focus on local public health authorities and less focus on officials in other sectors. Secondly, twenty-six health jurisdictions were excluded. Among the ten included, most interviewees represented central and especially southern regions; thus, experiences from northern communities are not adequately captured. Finally, the study does not address particular challenges of climate change adaptation where there are vulnerable groups. While these caveats are important, results enrich existing knowledge on climate change adaptation in the Ontario health sector and provide a basis from which to inform future action.

Climate change is on the radar of health officials concerned about impacts exacerbating existing health issues and/or creating new ones. Notably, heat, extreme weather and poor-air quality are high concerns; though risk perception cannot be generalized across Ontario as most respondents represented southern Ontario jurisdictions. Health officials acknowledge their unique role in climate change adaptation; this experience will need to be drawn upon to meet the challenges posed by climate change [15]. Adaptation in the public health sector will be a challenge in part due to limited understanding of the various ways in which it has and will affect health outcomes and public health resources and uncertainties in future climate vulnerability projections. Managing uncertainty will be challenging because public health resources are typically allocated to acute and widespread health concerns over potential future ones. These challenges must be met with proactive efforts on the part of health units in Ontario, and in other health jurisdictions in the face of climate change. Furthermore, the public health response must accept uncertainty to avoid financial and social costs of maintaining the status quo calling for a need to balance management of current and projected long-term climate related health risks [33,34].

Currently, there is difficulty in operationalizing existing standards on climate change. Ontario health departments seek ways to mainstream climate change but are constrained by limited resources and guidance. Specifically, health units are required to increase public awareness on health hazards associated with climate change; but information on climate-related health burdens is limited and existing mechanisms to promote awareness may be ineffective. Promoting awareness, although critical, will be insufficient to prevent climate change risks to human health. Public health activities will contribute to reducing climate related health risks, but capacity needs to be assessed iteratively via evaluations and research [35] as effective health warning systems may not translate to adequate responses and interventions locally [29]. Warning systems and interventions for heat and smog days, for example, differ across municipalities in Ontario [36] as best efforts are not yet standardized in health policies. Efforts to monitor burden of illness associated with climate change health vulnerabilities on local scales are currently lacking [3]. Climate change and health risk indicators can be identified via epidemiological studies and expanding empirical databases [37], but will require sustained funding and research expertise to accomplish.

Climate change may be best managed in health departments via an adaptation strategy that could guide planning and implementation, streamline efforts across the health department, and inform the need for resources and staff training to prepare effectively for climate change. In Ontario health departments, climate change considerations are limited to specific health hazards per health department; there is a need for climate change to be integrated across the wider range of environmental health and chronic disease prevention areas. This is because climate change may create public health risks that health departments and partners lack experience in managing. Local stakeholders need to plan for potential impacts based on knowledge of how climate change will affect the region and design programs accordingly. Furthermore, an adaptation strategy could help to raise awareness among managers and promote the integration of a climate change perspective into risk assessments [38]. Considering the various pathways in which climate change will impact health, adaptation strategies should be established using a community based framework [14,29,39]. Climate change health risk assessments require updated information on vulnerable groups that can be gained through multi stakeholder efforts and community partners, local officials and medical professionals can provide local information on vulnerable groups, intervention gaps and needs [40,41]. Community level adaptation enables resource sharing, synergizing goals and strategies increases adaptive capacity and fosters communication between health and municipal officials on the health risks who otherwise may not consider health factors and where institutional and communication barriers exist between them [39]. Community based forums facilitate resource sharing; resource needs in the form of technical skills, tools and knowledge should be shared across sectors to maximize adaptation success. In Ontario, barriers such as competing priorities and departmental silos have the potential to hinder implementation of inter-departmental adaptation goals. A particular challenge for communities in Ontario, especially areas expected to undergo large population increases will be in developing strategies for mitigation and adaptation. This is already a challenge; for example, public health adaptation and mitigation messages are contradictory when residents are encouraged to use less energy on smog days and to use their air conditioners if it is also extremely hot. Research is needed to identify win-win strategies, to avoid inefficient resource use and confusion.

This study suggests that public health adaptation requires higher levels of government effort, particularly provincial public health policies on climate change adaptation and the continued support from federal departments and agencies. The province needs to undertake a comprehensive climate change vulnerability assessment in the health sector to inform research and policy gaps. Provincial public health agencies need to adopt anticipatory adaptation strategies by investing in climate change research to inform effective policies on local public health adaptation efforts and ensure that climate change is integrated into health risk management. There is a need for higher resolution climate projection models, improved and standardized surveillance, and monitoring and early warning systems to manage climate related health risks. Ontario public health departments would benefit from a research center aimed at advancing environmental health surveillance and warning systems, to support local research pilot projects, epidemiological studies and to establish long-term monitoring systems to track climate change driven health outcomes and program and policy effectiveness to reduce those risks. Health department adaptation resources will be required for enhancing programs and will need to come from higher government sources. The province already supports the development of risk management tools to manage heat related risks and works closely with public health units to raise awareness of health hazards associated with Lyme disease [21]. Further efforts could capitalize on the recently released Ontario Climate Ready Adaptation Plan [21], which calls for enhanced provincial health policies on climate change. The provincial plan provides the impetus to allocate resources to climate change and health initiatives, a source to identify available health policy adaptation tools (Climate Modeling Collaborative) and to identify opportunities to mainstream public health considerations into adaptations occurring in other sectors.

Sustained funding is also needed to support policies for all climate change health vulnerabilities. Federally funded and locally targeted adaptation programs should be continued as their support has greatly contributed to advancing adaptation at the local level. Federal level efforts should continue to assist in the development of surveillance and monitoring tools, adaptation best practices and downscaling climate change models in order to be used in practice at regional or municipal levels. Ontario health officials need evidence concerning whether existing efforts are effective and standardized best practices for climate change health risk prevention and responses. Ontario officials have indicated that awareness raising and education are key roles for public health departments on adapting to climate change. The call for more effective risk communication for heat warning systems to prevent heat related deaths [41,42] has been addressed by Health Canada in their Heat Alert and Response System research initiatives; however, further research is needed for other health issues. Ontario health officials have indicated that provincially mandated programs may not be sufficient for adapting to climate change and that more information is needed. Informational and financial support from federal departments and agencies are necessary to compliment provincial efforts and to address existing knowledge gaps. Federal support has facilitated health adaptation via the Ontario Regional Adaptation Collaborative (Natural Resources Canada) in funding research and pilot projects (Health Canada and Public Health Agency of Canada) and informing public health policy. One way to ensure that federal funding is sustained over the long-term and that efforts are coordinated across health jurisdictions is for Canada to develop a climate change adaptation strategy; similar to adaptation plans implemented in other countries.

## Competing interests

No author had any financial conflict of interest. No author was or is employed in the Ontario Public Health sector. At the time of researching, writing and submitting the manuscript, Jaclyn Paterson was employed at McGill University; at the time of publication, she was employed at Health Canada. Peter Berry was an employee at Health Canada during the full duration of the project.

## Authors’ contributions

JP took the lead in conceptualizing and designing the study, conducted interviews, performed literature searches, and analyzed and interpreted data. JF, LB, JH, JH and AL made substantial contributions to the study design, interpreting data and critically drafting and revising the final manuscript. PB contributed substantially to the study design and in critically drafting and revising manuscript versions. All authors read and approved the final manuscript.

## Pre-publication history

The pre-publication history for this paper can be accessed here:

http://www.biomedcentral.com/1471-2458/12/452/prepub

## Supplementary Material

Additional file 1 Note that electronic Additional files (word document), entitled: *BMC Public Health Additional files- Adaptation to Climate Change in the Ontario Public Health Sector* are available which summarize responsibilities pertinent to adaptation in health and health relevant sectors in Ontario. Click here for file

Additional file 2 Description of inclusion and exclusion criteria for selection of regional health jurisdiction. Click here for file

Additional file 3 Interview participants. Click here for file

Additional file 4 Information pertaining to regions included in interviews and the number of interviews per region. Click here for file

Additional file 5 Full survey instrument. Click here for file

Additional file 6 Codebook for identifying key themes in interview responses. Click here for file

Additional file 7 Additional representative quotes. Click here for file
