# Supplementary material for: Adaptation to climate change in the Ontario public health sector
Source: BMC Public Health. 2012 Jun 19;12:452. doi: 10.1186/1471-2458-12-452 (PMC3418204; doi:10.1186/1471-2458-12-452)
Supplement: Additional file 1 — Note that electronic Additional files (word document), entitled: BMC Public Health Additional files- Adaptation to Climate Change in the Ontario Public Health Sector are available which summarize responsibilities pertinent to adaptation in health and health relevant sectors in Ontario. [file 1471-2458-12-452-S1.doc]

Additional file 1

Note that electronic Additional files (word document), entitled: *BMC Public Health Additional files- Adaptation to Climate Change in the Ontario Public Health Sector* are available which summarize responsibilities pertinent to adaptation in health and health relevant sectors in Ontario.

| **Public Health**   - Health Hazards (indoor and outdoor air quality, extreme weather, vector borne diseases and climate change): Undertake work to prevent or reduce the burden of illness from health hazards in the physical environment. - Water safety: Undertake work to prevent or reduce the burden of water-borne illness related to drinking water; to prevent or reduce the burden of water-borne illness and injury related to recreational water use. - Food safety: Undertake work to reduce the burden of food borne illness. - Infectious diseases: Undertake work to prevent or reduce the burden of infectious diseases of public health importance - Chronic Disease Prevention and UV radiation: Undertake work to reduce the burden of preventable chronic diseases of public health importance - Public health emergency preparedness: Undertake work to enable and ensure a consistent and effective response to public health emergencies and emergencies with public health impacts. |
| --- |
| **Emergency management**   - Involved in planning and preparing for emergencies in the community, organizing and coordinating emergency efforts, response planning, and training, responding to emergencies and direct outreach to the public. This includes emergencies associated with climate change, specifically extreme weather; for example, floods, storms, extreme heat or cold episodes. |
| **Planning**   - Involved in land-use planning, urban design (designing streets, parks and open spaces, building location / organization and shape), housing, community services and the environment, heritage preservation, transportation (walking, cycling, subways and streetcars), growth planning development and review, bylaws and zoning issues. Planners may be directly involved with climate change plans and / or initiatives that are relevant to addressing health impacts associated with climate change via regional or city plans and strategies (e.g. mainstreamed into water quality and quantity, land use planning to promote green urban designs and clean air). |
| **Environment and Conservation**   - Involved in protection and preservation of land, water and air, integrating societal and natural / environmental interests in the development and implementing of environmental plans. This includes how climate change affects the environment (land, water, air) and society. Included are water and waste-water management activities, drinking source water protection plans and programs. Conservation: authorities involved in watershed planning, flood risk mapping, warnings, regulation of land developments (restrict development in flood prone zones, unstable lands), public awareness and outreach. Climate change is implicated in some planning and outreach activities. |
