# Supplementary material for: Adaptation to climate change in the Ontario public health sector
Source: BMC Public Health. 2012 Jun 19;12:452. doi: 10.1186/1471-2458-12-452 (PMC3418204; doi:10.1186/1471-2458-12-452)
Supplement: Additional file 2 — Description of inclusion and exclusion criteria for selection of regional health jurisdiction. [file 1471-2458-12-452-S2.docx]

Additional file 2

A description of inclusion and exclusion criteria for selection of regional health jurisdiction.

| **Category** | **Inclusion criteria** | **Exclusion criteria** |
| --- | --- | --- |
| Geographic region | Range of municipalities must include at least one representative municipality from southern and central Ontario | Any municipality such that the range of municipalities does NOT include at least one representative sample of municipalities covering the southern and central regions of Ontario |
| Population characteristics | Range of municipalities included must represent urban and rural, high and low income, mixture of ethnic and cultural diversity, mixture of densities and demographics. All municipalities were expected to include some mixture of immigrations, low socio-economic status, elderly, children, and individuals with pre-existing or chronic diseases. | Any municipality such that the range of municipalities that are ALL urban or ALL rural based populations or ALL high income or ALL low income etc. |
| Level of climate change adaptation awareness and preparation | Municipality has a climate change adaptation plan OR an explicit climate change adaptation initiative planned for the future, currently in progress or that has been completed or fully implemented. The public health department must be involved in the planning and / or implementation of the climate change action plan or initiative. *OR Public* health department is involved in one or more initiatives (e.g. programs and / or services and / or projects and / or policies etc.) that explicitly address climate change. Climate change does not have to be the primary or sole motivation for planning or implementing the initiative.  Awareness had to be explicitly focused on climate change AND one or more of the health vulnerabilities associated with climate change. These include: Extreme heat or cold; Extreme storm; Flood; Drought; Air quality; UV radiation; Wildfire; Food / water / vector-borne diseases; Food / water quality and quantity.  Climate change adaptation initiatives were considered to be specific actions that address one or more of the health vulnerabilities associated with climate change. Examples of relevant initiatives included: Research projects; Impact, vulnerability, risk or adaptive capacity assessments; Policies, plans or conceptual / informational tools; Modeling or technical tools; Monitoring and surveillance; Stakeholder networking; Public awareness and outreach; Alerts and warnings; Departmental development / program evaluations; Infrastructure; Legislation. | No evidence that the municipality is explicitly aware of actively involved in or concerned about health risks associated with climate change.  No evidence that the public health department has been is or will be involved in any explicit climate change adaptation relevant initiatives. |
