# Supplementary material for: Adaptation to climate change in the Ontario public health sector
Source: BMC Public Health. 2012 Jun 19;12:452. doi: 10.1186/1471-2458-12-452 (PMC3418204; doi:10.1186/1471-2458-12-452)
Supplement: Additional file 3 — Interview participants. [file 1471-2458-12-452-S3.docx]

Additional file 3

Interview participants

| **Category** | **Inclusion criteria** | **Exclusion criteria** |
| --- | --- | --- |
| Jurisdiction | Federal level with Ontario jurisdiction (public health)  Ontario provincial (public health)  Ontario regional or municipal (public health and other sectors) | All other Canadian Provinces and Territorial jurisdictions |
| Employment type | Currently employed as a government or quasi government official | Non-government employee |
| Sector | Currently employed in one of:   - Public health - Planning - Emergency management - Water and utilities - Environment and conservation | Physical infrastructure  (and all other sectors) |
| Level and nature of responsibilities | Management and / or director and / or officers and / or persons in senior level positions. Included are policy analysts and environmental health specialists  Managerial role over program(s) and / or service(s); policy and / or program and / or service planning; research and / or policy analyst  Responsible or actively take part in inter-agency collaborations, communications, participations (i.e. interactions with other government and / or non-government agencies at the regional and / or provincial and / or federal level)  Awareness and familiarity of regional / municipal provincial and federal legislation, standards, policies and protocols relevant to persons work done | Staff who are NOT involved in decision making, policy or program planning, overseeing programs and services across one or more relevant program areas  Staff who do NOT participate in inter-agency meetings, partnerships, collaborations at the regional, provincial and / or federal level |
| Stimulus for carrying out responsibilities | Work carried out must implicitly or explicitly reduce risks / minimize / address one or more public health vulnerabilities associated with climate change. These include:   - Extreme heat or cold - Extreme storm - Flood - Drought - Air quality - UV radiation - Wildfire - Food / water / vector-borne diseases - Food / water quality and quantity | Work carried out does NOT implicitly or explicitly address any of the public health vulnerabilities associated with climate change. |
