# Supplementary material for: Adaptation to climate change in the Ontario public health sector
Source: BMC Public Health. 2012 Jun 19;12:452. doi: 10.1186/1471-2458-12-452 (PMC3418204; doi:10.1186/1471-2458-12-452)
Supplement: Additional file 4 — Information pertaining to regions included in interviews and the number of interviews per region. [file 1471-2458-12-452-S4.docx]

Additional file 4

Information pertaining to regions included in interviews and the number of interviews per region.

| **Health Unit** | **Ontario Sub-Region** | **Population**  **(2007)^[[1]](#footnote-2)^** | **Municipal jurisdiction^[[2]](#footnote-3)^** | **Number of Municipalities** | **Peer group^[[3]](#footnote-4)^** | **No. Interviewees of PH^[[4]](#footnote-5)^ and NPH** |
| --- | --- | --- | --- | --- | --- | --- |
| 1. Sudbury District | North East | 198,265 | District of Greater Sudbury | 19 | Sparsely populate urban rural mix | 1 PH; 1 NPH (Conservation Authority) |
| 2. Ottawa | Eastern | 846,169 | City of Ottawa | 1 | Urban centers | 3 PH; 1 NPH (planner) |
| 3. Toronto | Central East | 2,651,717 | City of Toronto | 1 | Metro Center | 10 PH; 2 NPH (1 planner, 1 environment official) |
| 4. Peel | Central East | 1,296,505 | Region of Peel (upper tier) | 3 | Urban centers | 4 PH; 2 NPH (1 emergency management official, 1 Planner); 3 lower-tier municipal officials each in Caledon, Brampton and Mississauga (environment officials) |
| 5. York | Central East | 975,906 | York Region (upper tier) | 9 | Urban centers | 6 PH; 2 NPH (1 planner, 1 environment official) |
| 6. Halton | Central East | 468,980 | Halton Region (upper tier) | 4 | Urban centers | 1 PH |
| 7. Peterborough County-City | Central East | 133,583 | Peterborough County (upper tier) | 9 | Urban / rural mix | 1 PH |
| 8. Norfolk and Haldimand | Central West | 111,684 | Haldimand-Norfolk | 2 | Mainly rural | 1 PH |
| 9. Niagara Region | Central West | 433,946 | Regional Municipality of Niagara (upper tier) | 12 | Urban / rural mix | 3 PH; 3 NPH (2 planners, 1 environment official) |
| 10. Windsor-Essex County | South West | 403,797 | Windsor-Essex | 9 | Urban centers | 1 PH; 4 NPH (3 emergency management officials, 1 environment official) |

1. Number of individuals residing in the health unit based on population estimates [↑](#footnote-ref-2)
2. There are 444 local municipalities in Ontario. Lower-tiered municipalities exist when there is another level of municipal government which provides services to residents (referred to as “upper tier” municipalities). Where there is only one level of municipal government in an area, it is called a single tier municipality. [↑](#footnote-ref-3)
3. Urban – rural characteristics: Mainly rural regions: high proportion of people recently moved to or within these regions since 1996; average percentage of Aboriginal population, moderate population growth; Sparsely populate urban rural mix: Sparsely populated urban rural mix average percentage of aboriginal population, negative population growth; Urban-rural mix: urban-rural mix from coast to coast; average percentage of aboriginal population, low male population, slow population growth from 1996-2001; Urban centers: mainly urban centers with moderately high population density, low percentage of government transfer income, rapid population growth from 1996-2001; Metro-center: Largest metro centers with an average population density of 3,943 people per square kilometer, low aboriginal population, moderate percentage of government transfer income, high female population [↑](#footnote-ref-4)
4. PH = Public health official; NPH = Non-public health official [↑](#footnote-ref-5)
