# Supplementary material for: Adaptation to climate change in the Ontario public health sector
Source: BMC Public Health. 2012 Jun 19;12:452. doi: 10.1186/1471-2458-12-452 (PMC3418204; doi:10.1186/1471-2458-12-452)
Supplement: Additional file 5 — Full survey instrument. [file 1471-2458-12-452-S5.docx]

Additional file 5

The full survey instrument.

| **Theme** | **Guiding Questions** |
| --- | --- |
| **General information**  *Aim: to obtain contextual information on respondent’s area(s) of responsibility (including degree that health is explicitly integrated into non-public health departments at the regional / municipal level), number of staff, population base, and governance structure* | - What is your role at your [*public health department*] [*agency*]? Please describe the program areas that you [*are involved with at your public health department*] [*specifically work in*]? How long have you been working with your [*public health department*] [*agency*]? - How many people work at your [*public health department*] [*agency*]? [*Posed to regional officials*] Sub-question: would you please comment on the general population (demographics, ethnical diversity, rural versus urban) in your region? - [*Posed to regional public health officials*] Would you please describe the governance structure of your public health unit relative to others in the province? What makes your structure similar and different from the governance structure of other health units in Ontario? [*Posed to regional non-public health officials*] To what extent does your work directly or indirectly involve public health goals, initiatives and / or communications with public health officials? Note that this question addresses the extent to which public health is included in your mandate; whether it be to collaborate with public health officials and / or to integrate public health initiatives into your plans and programs. |
| **Priority of climate change and health risks of concern**  *Aim: to gain an understanding of the influence of top-down governance and its affect on the attention given to climate change adaptation at lower government levels and / or the priority given to climate change within a department or division relative to other topics* | - [*Posed to federal and provincial health officials*] In the current federal government, how would you describe the priority given to climate change (adaptation) at the national level in Canada? Sub-question probe: would you say it is low, medium or high for the federal government? Sub-question probe: does the level of priority given to climate change (adaptation) at the federal level affect the level of priority given to climate change for your agency? - [*Posed to Ontario provincial government health officials*] How would you describe the priority given to climate change at the provincial level in Ontario? Sub-question probe: would you say that it is a low, medium or high priority for the current Ontario Provincial Cabinet? Sub-question probe: does the level of priority given to climate change at the provincial cabinet affect the level of priority given to climate change for your agency? - Within your [*public health department*] [*agency*], how would you describe the level of priority given to climate change (adaptation) relative to other [*health issues*] [*issues*] [*plans / policies and / or programs*] in your region both (a) currently and (b) in the future? Note that answers will vary depending on each individual’s program area, personal experience and perspective. - Experts have identified many climate change hazards that impact health. [*Please choose 3-5 vulnerabilities from the following list* *that you think* *pose the greatest health risk in your region. Follow-up: Can you explain why they are important risks in your area?*] [*Would you be able to describe or discuss how one or more of these risks is integrated into your agencies plans / policies / programs?]* Extreme heat; Extreme cold; Storms; Floods; Drought; Air Quality; UV Radiation; Wildfires; Food-borne diseases; Food quality and quantity; Water-borne diseases; Water quality and quantity; Vector-borne diseases. Note that that answers will vary depending on each individual’s program area, personal experience and perspective. When answering, please consider both the (a) source climate hazard and the (b) health implication that you are concerned with as a result of the climate hazard. |
| **Characterization of Adaptation Actions**  *Aim: to understand the process of adaptation in terms of key components of the action which could include one or more of: motivation, process, resources, stakeholders, targeted population, monitoring, challenges and drivers* | Please identify and describe any health initiatives (plans, policies and/or programs) in your jurisdiction that were/are : a) motivated primarily by cc, b) established in part by cc, or c) relevant to addressing health impacts of cc, but were / are not motivated by cc at all. This question refers mainly to c adaptation initiatives, however, you are free to discuss mitigation initiatives as well. In your description, please include the motivation for taking action, stakeholders involved, and challenges and facilitators of progress you have come across during the planning and implementation process. Topic probes: Motivation (cc impact observed or anticipated, mandated or autonomous); Process (Adaptation strategy, policy in place, program design, legislation (Y/N)); Resources (Technical / informational, internal/external, non-gov’t / gov’t); Stakeholders (Federal, provincial, local, non-gov’t, inter-sectoral, public); Targeted population (all persons, vulnerable groups identified or prioritized); Monitoring (tracking progress, consideration of changing program costs & resources); Challenges and Drivers (Institutional, gov’t, communication, leadership, resources). |
| **Adequacy of top-down support to facilitate local climate change adaptation**  *Aim: to assess if & how federal &provincial gov’t regulations & other guidelines are facilitating regional/ municipal cc adaptation, and if more top-down instruction or support is needed* | Would you please comment on the adequacy of legislation, protocols and / or guiding documents at the federal and / or the provincial level to guide [*your agency's*] [*regional and / or municipal governments of Ontario*] ability to address the health impacts of climate change? Specifically, I am interested in the adequacy of the [*Ontario Public Health Standards*] [*Provincial ministry standards or protocols*] and Federal government guiding documents. |
| **Perceptions of Adaptive Capacity**  *Aim: to assess the adequacy of partnerships or collaborations (inter & intra jurisdictional, inter-departmental, public-private) and resources (existing programs, services, resources and staff) for the respondents agency to be able to adapt to health risks of climate change.* | - [*Posed to regional public health officials*] Would you please comment on the adequacy of intra-jurisdictional collaborations to guide your agency's ability to address the health impacts of climate change? Intra–jurisdictional refers to collaborations across divisions within your public health unit. - Would you please comment on the adequacy of inter-jurisdictional collaborations to guide your agency's ability to address the health impacts of climate change? Can you describe inter-collaborations in terms of partnerships between your [*public health department*] [*agency*] and (a) [*other*] regional and / or in municipal departments [*public health department*] (b) federal and / or provincial departments / ministries and (c) private or non-government organizations? - Would you please comment on the adequacy of existing programs and services, resources and staff to guide your agency’s ability to address health impacts of climate change? [*Posed to regional non-public health officials*] Do you have suggestions on how your agency must evolve to prepare for impacts of climate change in the future? |
| **Roles and responsibilities**  *Aim: to assess how different respondents perceived the roles and responsibilities for climate change adaptation spread across levels of government, different government sectors, between private and public sectors and the general public.* | - Would you please comment on the role of public health departments of Ontario in preparing for health impacts of climate change? Also, do you have suggestions on how [*your*] public health department[*s*] must evolve to prepare for health impacts of climate change in the future? - How do you presently see roles and responsibilities for climate change adaptation shared among levels of government and between government and non-governmental entities (e.g. NGOs, academia, private industry and the public)? What about in the future?   - [*Posed to all regional officials*] Sub-question probe- Please comment on the ways in which the upper levels of government have helped or could help you better prepare for the health impacts of climate change. I.e. in what ways do higher levels of government assisting or supporting you and / or representing your interests?   - [*Posed to provincial and federal health officials*] Sub-question probes - Would you comment on the notion that climate change adaptation is primarily a local level responsibility? What is the role of your jurisdiction in addressing public health impacts of climate change in comparison to other jurisdictions? [*Posed to provincial health officials*] how do you see your ministries role and that of other ministries in addressing health impacts of climate change? If you are an official in a federal department or agency, how do you see your department’s role and that of other federal departments or agencies in addressing health impacts of climate change? Would you be able to comment on the notion that Ontario would be better equipped to address health impacts of climate change if the federal government established a national adaptation strategy? To what degree is your agency responsible for providing tangible guiding principles for regional and / or local governments? Note: this question has been posed to assess the level of direction that ministries are mandated to provide for regional governments. |
| **Suggestions for the future**  *Aim: to identify the opportunities for agencies to be better able to adapt to health risks of cc.* | Would you please suggest three things that need to change in order for your [public health department] [agency] to be better prepared to address the public health challenges of climate change? Please provide an explanation for each. |
