# Supplementary material for: Adaptation to climate change in the Ontario public health sector
Source: BMC Public Health. 2012 Jun 19;12:452. doi: 10.1186/1471-2458-12-452 (PMC3418204; doi:10.1186/1471-2458-12-452)
Supplement: Additional file 6 — Codebook for identifying key themes in interview responses. [file 1471-2458-12-452-S6.docx]

Additional file 6

The codebook for identifying key themes in interview responses.

| Category | Definition |
| --- | --- |
| **1. What is your role at your [*public health department*] [*agency*]? Would you please describe the program areas that you [*are involved with at your public health department*] [*specifically work in*]?** | |
| Roles | - [Public health only] Medical officer of health: responsible to the board of health for the management of the public health programs and services, directs staff of the board of health (who are responsible to the MOH) if their duties relate to the delivery of public health programs or services, has authority that is limited to the health unit served by the board of health. Attends board of health meetings. - Program manager: responsible for overseeing one more of the topic areas relevant to climate change. Administrators of programs, manages or oversees implementation of program components. This includes program managers of specific programmatic activities or directors of more than one programmatic area. - Coordinator or specialist: an official who holds a non-managerial position but a specialization in one or more particular areas relevant to public health and climate change. Individuals coordinate activities and advise on program components. - Research and Policy analyst: Policy support and informing programs related to public health and climate change. Officials involved in research, education, policy development and advocacy; monitoring and responding to emerging issues relevant to climate change and public health, providing technical, policy and educational support to city (or municipality, or regional council) staff. |
| Program / work area: Regional public health departments | - Regional Public Health officials - Health Hazards (indoor and outdoor air quality, extreme weather, vector borne diseases and climate change): Undertake work to prevent or reduce the burden of illness from health hazards in the physical environment. - Water safety: Undertake work to prevent or reduce the burden of water-borne illness related to drinking water; to prevent or reduce the burden of water-borne illness and injury related to recreational water use. - Food safety: Undertake work to reduce the burden of food borne illness. - Infectious diseases: Undertake work to prevent or reduce the burden of infectious diseases of public health importance - Chronic Disease Prevention and UV radiation: Undertake work to reduce the burden of preventable chronic diseases of public health importance - Public health emergency preparedness: Undertake work to enable and ensure a consistent and effective response to public health emergencies and emergencies with public health impacts. - Source: Ontario Public Health Standards 2008 <http://www.health.gov.on.ca/english/providers/program/pubhealth/oph_standards/ophs_mn.html> |
| Program / work area –  Regional:  Emergency management, Planning, Environment and Conservation  Federal and Provincial Public Health | - Emergency management: involved in planning and preparing for emergencies in the community, organizing and coordinating emergency efforts, response planning, and training, responding to emergencies and direct outreach to the public. This includes emergencies associated with climate change, specifically extreme weather; for example, floods, storms, extreme heat or cold episodes. - Planning: involved in land-use planning, urban design (designing streets, parks and open spaces, building location / organization and shape), housing, community services and the environment, heritage preservation, transportation (walking, cycling, subways and streetcars), growth planning development and review, bylaws and zoning issues. Planners may be directly involved with climate change plans and / or initiatives that are relevant to addressing health impacts associated with climate change via regional or city plans and strategies (e.g. mainstreamed into water quality and quantity, land use planning to promote green urban designs and clean air). - Environment and Conservation: involved in protection and preservation of land, water and air, integrating societal and natural / environmental interests in the development and implementing of environmental plans. This includes how climate change affects the environment (land, water, air) and society. Included are water and waste-water management activities, drinking source water protection plans and programs. Conservation: authorities involved in watershed planning, flood risk mapping, warnings, regulation of land developments (restrict development in flood prone zones, unstable lands), public awareness and outreach. Climate change is incorporated into planning and outreach activities. - Federal health: involved in climate change and public health research, development of policies, guides, tools and / or best practices in public health adaptation to climate change. - Provincial health: involved in creating policies, legislations, standards and / or protocols and support for public health departments in Ontario. - Source: *Public Health Agency of Canada:* [*http://www.phac-aspc.gc.ca/index-eng.php*](http://www.phac-aspc.gc.ca/index-eng.php); *Climate Change and Health Office:* [*http://www.hc-sc.gc.ca/ewh-semt/climat/index-eng.php*](http://www.hc-sc.gc.ca/ewh-semt/climat/index-eng.php); *Ministry of Health Promotion and Sport:*[*http://www.mhp.gov.on.ca/en/*](http://www.mhp.gov.on.ca/en/); *Ontario Agency for Health Protection and Promotion:*[*http://www.oahpp.ca/*](http://www.oahpp.ca/) |
| **2. How long have you been working with your [*public health department*] [*agency*]?** | |
| (a) in total and / or  (b) in your current position | Number of years as a full time employee at the agency and / or within the specific branch.  1 = 0-4 yrs; 2 = 5-9 yrs; 3 = 10-14 yrs; 4 = 15-20 yrs; 5 = 20 or more yrs |
| **3. How many people work at your [*public health department*] [*agency*]?** | |
| (a) overall organization and/or (b) specific branch | Number of full time equivalents working at the overall agency and / or within the specific branch. The numbers of staff at each agency was identified using online resources e.g. websites, where available. |
| **4. [*Posed to regional public health officials*] Would you please describe the governance structure of your public health unit relative to others in the province? What makes your structure similar and different from the governance structure of other health units in Ontario?** | |
| Autonomous board of health | - Independent of council. Operates separately from the administrative structure of their municipalities. Board members include a mix of provincial representation, regional and / or municipal councillors and / or volunteers from the community who are interested in public health - *Source:* Ontario Ministry of Health and Long-term Care website: <http://www.health.gov.on.ca/> |
| Non-autonomous board of health is | Integrated into council. Board members are councils of single tier or double tiered municipalities.  *Source:* Ontario Ministry of Health and Long-term Care website: <http://www.health.gov.on.ca/> |
| **5. Within your [*public health department*] [*agency*], how would you describe the level of priority given to climate change (adaptation) relative to other [*health issues*] [*issues*] [*plans / policies and / or programs*] in your region both (a) currently and (b) in the future?** | |
| Low | - Climate change is / will be not a priority. It is a low priority compared to other issues; it is not mentioned explicitly in most items and is rarely implicitly considered. Staff members do not consider climate change when carrying out services to the public. |
| Med | - Climate change is / will be somewhat of a priority; it is not the top priority, but there is awareness of climate change risks. Climate change is implicitly driving some of the work and on the minds of some of the staff for certain programs. |
| High | - Climate change is / will be among the most pressing issues. It is a broad priority for long-term planning; it is an explicit driver in some work carried out. Proactive effort is taken to prepare for climate change. |
| **6. Experts have identified many climate change hazards that impact health. [*Please choose 3-5 vulnerabilities from the following list* *that you think* *pose the greatest health risk in your region. Follow-up: Can you explain why they are important risks in your area?*] [*Would you be able to describe or discuss how one or more of these risks is integrated into your agencies plans / policies / programs?]*** | |
| Extreme heat or cold | - Very high or low temperatures that cause physical or mental stress. |
| Extreme weather / Storms & Flooding | - Extreme wind and rain storms / heavy rain (not “flood”), tornadoes, freezing rain, thunderstorms, lightning and hurricanes. “Extreme weather” is stated. - Storms associated with severe rain fall that lead to floods. Concern over physical health impacts from mould growth in homes. “Floods” is stated. - *Includes indoor air quality*. |
| Outdoor Air quality | - High ground-level ozone and particulate matter outside, high smog, allergens and pollen, concern over poor air quality and respiratory health and community wellbeing |
| UV radiation | - Ultraviolet radiation exposure, increased temperatures and ozone depletion, concern over increased risk of skin cancer |
| Wildfires | - Naturally caused forest fires leading to poor air quality. Concern over increased respiratory illness. |
| Food-borne contamination: Food-borne diseases / Food quality and quantity | - Livestock stressed by temperature are more likely to become ill, which would lead to a greater risk of meat contamination during processing, climate-related changes in wild bird/animal populations could cause new food-borne pathogens to emerge, power outages associated with high energy demands (heat waves) or extreme weather could cause refrigeration failure, climate-caused changes in human behaviour that increase risk of food spoilage (e.g. more BBQs), changes in marine ecology (eutrophication and toxin-producing species) that lead to human toxicity through ingestion of contaminated fish / shellfish |
| Water-borne contamination: Water borne diseases / water quality and quantity | - Warmer temperatures leading to emergence and spread of water borne pathogens, (*Cryptosporidium*, *Campylobacter*, *E. Coli*, toxic microalgae (associated with eutrophication), blue-green algae). Water may be contaminated through increased overland flow (or storm water runoff in urban environments) due to increased precipitation. Concentrated contaminants in water during droughts. Concern over increased enteric diseases and illness from recreational exposure (e.g. wound infections due to swimming). - *Includes drought. Note that some respondents selected water contamination but associated it with storms and floods.* |
| Vector and rodent borne diseases | - Climate change may alter the geographical distribution, the seasonality, and the incidence of vector-borne / zoonotic diseases. This includes the emergence of diseases that are currently thought to be rare or exotic to Canada. Concern over emergency of mosquito borne illnesses: West Nile virus, Western and eastern equine encephalitis, Dengue fever and / or malaria in the community. Concern over emergence of the tick borne Lyme disease |
| **7. Would you please identify and describe any [public health relevant adaptation] initiatives (plans, policies and / or programs) in your jurisdiction, that were / are either (a) explicitly motivated primarily by climate change (b) established in part by climate change (c) relevant to addressing health impacts associated with climate change, but not motivated at all by climate change?** | |
| Primary or Secondary Motivation  Bottom up or Top down | - Primary: initially driven on the premise of addressing climate change or climate change is a strong explicit consideration for improving or changing the initiative or program - Secondary: driven initially by factors(s) unrelated to climate change; however, climate change was implicitly considered during the planning and / or implementation process. Also, initiatives that could address climate change but climate change is not considered at all during any stage of the planning and implementation process. |
|  | - Bottom-up: locally driven, not mandated to carry out the initiative by provincial or federal legislation. - Top-down: Mandate to carry out the initiative by provincial or federal legislation. |
| Stakeholders | - Individuals, groups or agencies involved in the planning and / or implementation process. Stakeholder categories included: - Public Health Unit: “an official health agency established by a group of urban and rural municipalities to provide a more efficient community health program, carried out by full time, specially qualified staff”. <http://www.health.gov.on.ca/english/public/contact/phu/phu_mn.html> - Regional / municipal civil department: division of the regional or municipal organization and could include one or more departments representing various sectors. Examples include: water and waste water, parks and recreation, planning, environment, social housing, transportation and utilities. For two-tiered government structures, departments could be part of an upper tier regional department or a lower tier municipality. - Federal government: Canadian government departments and agencies and representatives and arm’s length federal government entity. Examples include Health Canada (e.g. climate change and air quality initiatives), Environment Canada (e.g. extreme weather, climate change information), Natural Resources Canada, Canadian Food Inspection Agency (food recalls), Canadian Climate Change Scenarios Network (climate change scenarios), Public Health Agency of Canada (vector-borne disease and climate change research) - Provincial government: Province of Ontario government ministries and representatives and arms length provincial government agencies. An example of an arm’s length provincial agency is Ontario Agency for Health Protection and Promotion (<http://www.oahpp.ca/>) - Conservation Authority: In Ontario, there are 36 conservation authorities which are autonomous corporate bodies responsible for the management of water and other renewable natural resources in the province of Ontario and legislated under the Conservation Authorities Act. (<http://www.conservation-ontario.on.ca/>). - Non-government: not for profit, voluntary, non-state, citizens associations or independent institutions and think tanks. - Professional association, private entity, academic or institutions (e.g. Universities, Colleges, Institute of Catastrophic Loss Reduction). The category includes for profit, private businesses, corporations or consultants and un-associated individuals (families and individuals representing the public in general). - Adapted from Lesnikowski et al. 2011 (in submission) |
| Type of Action | - Research: Studies undertaken to identify health risks associated with climate change in the community; for example, burden of illness studies and / or research to identify thresholds and / or triggers for issuing health risk, effective response strategies. |
|  | - Risk / impact / vulnerability assessment: Standard risk assessments to identify and prioritize health hazards in the community that are in part driven by one or more of the climate change health vulnerabilities. Included are Hazard Identification and Risk Assessments (HIRA) carried out by public health emergency managers. This includes steps to better identify who is vulnerable in the community, what ways the community will be impacted and the general risks posed. Note assessments do not have to explicitly mention climate change. Included are developments of baseline assessments of community response capacity and vulnerabilities to one or more climate change health vulnerabilities and / or assessment of the efficiency and effectiveness of existing processes, programs, projects and activities that could contribute to reducing these risks. |
|  | - Planning and Conceptual tools: strategic development or policy documents created to guide current or future actions where one or more climate change health vulnerabilities are addressed. Examples include extreme weather response plans (extreme heat, cold) and emergency response plans. Also included are regional municipality official plans, climate change action plans, adaptation strategies, climate change adaptation discussion papers and action plans, and / or any other planning or policy documents summarizing short term and long term goals and actions that are relevant to addressing health impacts of climate change. |
|  | - Modeling and technical tool: Climate scenarios or predictive or illustrative modelling and mapping programs or tools that are explicitly used to inform climate change adaptation and / or to help inform programs that address public health threats associated with climate change (e.g. groundwater modeling, use of GIS in flood risk management, recreational water-quality modeling) were important components of the initiative. Note the modeling effort does not have to include future climate projections in designing and utilizing models. Models used to improve understanding of an existing or predicted health hazard and to inform programs and policies. Examples include air quality modeling. |
|  | - Surveillance and monitoring: important components of the initiative involved regularly monitoring or surveying public health risks posed by climate, weather or environmental health hazards. Examples include surveillance of vector populations, monitoring health outcomes from extreme weather events, monitoring climate and weather reports and forecasts (e.g. from Environment Canada or other private weather and / or extreme event forecasting sources) for monitoring in highly dense urban centers, testing water quality at beaches or private wells, scanning vulnerable groups during extreme heat events, monitoring federal or provincial weather reports. Also included are (a) identifying vulnerable populations or sub-groups and / or registries of vulnerable individuals who require assistance in an extreme weather event or emergency and / or who are identified as vulnerable to one or more climate change health vulnerabilities (e.g. rural communities on private wells may be more vulnerable to water-borne disease contamination proceeding an extreme rain event, outdoors workers who may be more vulnerable to extreme heat episodes, hikers or persons who visit or who are living in close proximity to sites known to be endemic for one or more vector-borne diseases like Lyme Disease or West Nile Virus) (b) monitoring and reporting of disease, injury, mortality incidence;, burden of illness associated with one or more climate change health vulnerabilities (c) establishing systems for reporting health impacts of climate related health outcomes (e.g. enhance quantitative data on short term and longer term health impacts of extreme weather events like floods and storms, extreme heat or cold, as examples) and (d) monitoring perceptions of the public or effectiveness of messaging to the public on one or more climate change and health risks (e.g. surveys distributed to the public to determine if health messages regarding protective measures against UV radiation, extreme heat, being bitten by a mosquito or tick or other vector harbouring dangerous pathogens) are received and what actions were taken in response if any). |
|  | - Stakeholder networking and partnership building: Partnerships, collaborations and / or communications were important components of the initiative. Examples include memorandums of agreements across agencies, workshops, webinars, conferences and information and knowledge sharing venues. Building partnerships among regional departments and with municipalities and other higher level government jurisdictions (international, federal, provincial), non-governmental or environmental organizations and private / industry representatives. |
|  | - Public awareness and outreach and communication: - Advocacy, educational, promotional and outreach activities aimed at increasing knowledge, promoting awareness, of health risks associated with weather, climate or environmental hazards. Incentive programs and encouraging environmentally friendly behaviours and attitudes. For example, promoting and encouraging use of alternative fuels, incentive programs for citizens, households, communities and corporations to reduce emissions and energy consumption. Initiatives may include workshops or events involving the public and / or presentations to the public (e.g. public outreach campaigns that educate residents on the importance of growing food locally / local food markets, how to safely manage private wells in rural communities). - Alerts and Warnings and Medical Interventions - Communicating messages to raise awareness and notify the public, municipal departments / staff and medical professionals and health care workers when extreme weather (e.g. issuing flood, storm, extreme temperature or air quality and smog advisories), infectious diseases, water (e.g. boiled water advisories, informing the public of recreational water / beach closures) and food borne disease (e.g. food recalls, full disclosure of test results from quality inspections of food premises) that pose a health risk. Messages are disseminated via established advisory, alert or warning systems and are targeted at the public, residents, travellers / tourists and / or vulnerable sub-populations / sub-groups, city and / or community partners who oversee them (e.g. messaging to elderly homes, homeless shelters etc.). Early warning systems to public, municipal staff / regional staff, health professionals and others to inform them in advance of an anticipated health hazard associated with climate change or weather. Information and messages distributed include (a) details about the health risks associated with one or more climate change health vulnerabilities; (b) suggested actions to take prior to, during and / or after climate change health hazard is projected, forecasted or is occurring (c) general information on precautions to take to avert risk. Information is disseminated via website postings, telephone calls, faxes, and emails and / or News media, such as the Weather Network or other sources such as print (i.e. newspapers), television, internet or radio. - Medical interventions: An example includes scanning apartment buildings for vulnerable individuals, isolated seniors or persons without air conditioning and distributing bottled water to homeless persons during an extreme heat / heat wave episode. |
|  | - Departmental development and program evaluation: The creation or improvement of a municipal department to address public health threats associated with climate change or the evaluation of the effectiveness and efficiency of existing adaptation efforts were important components of the initiative. Review and monitoring of existing programs undertaken to assess usefulness of the adaptations in order to suggest areas for improvement. |
|  | - Infrastructure Development and Technology and Engineering: Improvements or alterations to infrastructure or the physical / built environment were important components of the initiative. For example, improving the urban design to reduce the heat island effect. This includes increasing green spaces, installation of roof top gardens, planting trees, shading conditions along streets and parking lots. Also included are vector control measures such as reducing breeding grounds for mosquitoes, ticks and other vectors (e.g. larvaciding catch basins for controlling West Nile Virus), upgrading water treatment, sewage and sanitation facilities to deal with more severe extreme weather, improved public transit systems and bicycle lanes to reduce traffic related pollution levels, and provisions during extreme heat episodes. These include: creating accessible shelters or air conditioned public facilities, extending hours of cooling facilities, providing accessible drinking fountains in outdoor public places during extreme heat episodes. |
|  | - Legislation: bylaws and other regional / municipal local level legally binding requirements were important components of the initiative. Examples include anti-idling bylaws, green-roof bylaw, standing water bylaws, land development or building guidelines, food inspection requirements, and food handling certification regulations. - Adapted from Human Health in a Changing Climate, 2008, and Chiotti et al. 2002 and typology proposed in Lesnikowski et al. 2011 (in submission). |
| Target population | - General population: Initiative targeted toward the general public, both urban and rural with no specification for a particular demographic or vulnerable group - Rural: It is specified that the initiative is primarily geared toward residents living in rural dwellings. Urban: It is specified that the initiative is geared toward urban or city dwellers mainly or only. - Vulnerable group: The initiative is targeted at a particular segment of the population considered higher risk or more vulnerable to weather and climate related variables. Vulnerable groups were divided into the following 7 categories: (a) elderly / seniors (b) persons with chronic or pre-existing disease (c) Indigenous groups (d) pregnant mothers, infants and children (e) persons of low socio-economic status or low income (f) persons whose first language and commonly spoke language is not English or French (g) other vulnerable group mentioned by interview participant. - Adapted from typology proposed in Lesnikowski et al. 2011 (in submission). |
| Challenges | - Limited Resources - Inadequate funding: Staff members have identified what needs to happen, but have had challenges securing adequate funding to plan and implement the initiative. - Inadequate technical support and informational resources: the initiative is limited by not having adequate technical tools, weather or climate data to predict future trends, or guidance documents and evidenced based examples of appropriate specific actions to address the climate related health hazard(s). The technical tools and or informational support tools may exist but are not accessible or may not yet exist (e.g. local level predictions of climate and weather in the future to be able to predict extreme events). |
|  | - Communication Barriers - Stakeholder identification and communication challenges: there was difficulty in identifying the appropriate players to plan and implement the initiative as well as establishing the communication mechanisms and venues to ensure progress on the initiative. Comments in this category included lack of public awareness and / or difficulty in communicating with the public. - Lack of local political will and buy in: there is recognition that action should be taken to address climate change and public health specific to one or more of the health vulnerabilities in question 6, but there has not been consensus from regional and / or municipal council. This could be due to competing economic or societal priorities. - Staff coordination and role allocation: communication venues are in place; however, there are difficulties in ensuring that all players attend discussions, are engaged and proactively progress toward fulfilling roles. There is difficulty also in identifying who should have which roles. |
|  | - Program sustainability - The short-term and / or long-term fate of an initiative is vulnerable due to current or foreseen uncertainties in one or more of local government support, secured funding, and level of consensus or pro-activeness on the part of regional or municipal staff to carry the initiative forward. |
|  | - Respondents did not mention any challenges or stated they had not experienced any obvious challenges as of yet. |
| Drivers or Enablers | - Local political will and buy in - The initiative would not have gone forward if it were not for interest and consensus driven pressure from regional or municipal council. |
|  | - Local funding - The initiative would not have gone forward if it were not for dedicated funding specific to the particular aspects of the plan, program, project, or policy. The dedicated funding was given in large part by a government body. |
|  | - Capitalizing resources for change - Innovative and proactive problem solving and use of existing resources: There was no political pressure from the government to initiate and carry out the initiative. The initiative would not have gone forward if it were not for: - Creative ways to bring people together for brainstorming, information and knowledge sharing (uniting relevant stakeholders including government, non-for profit groups, private corporations, professional associations or individuals, non-for profit representatives, researchers at conferences and / or the public at information, brainstorming and knowledge exchange venues, webinars, workshops, and the formation of task forces and committees), - identifying and seeking out key non-human (guidance and support and technical tools or funding) or human (University or college students, volunteers such as the public or non-governmental outreach groups such as Red Cross or Salvation Army) resources - proactive research and awareness raising on a particular public health and climate change issue in order to promote collective effort to lobby for change. |
|  | - Stakeholder engagement and involvement - The initiative would not have gone forward if it were not for collective and proactive efforts on the part of the various stakeholders involved in planning and / or implementing the initiative. All stakeholders recognize the importance of the initiative and have shown evidence of targeted and maintained effort to ensure progress. |
|  | - Local leader(s) - The initiative would not have gone forward if it were not for the actions of one or more key individuals either on regional or municipal council (e.g. city or town mayor), public health representative (e.g. the medical officer of health) or dedicated staff. |
|  | - Timing - The initiative would not have gone forward if it were not for one or more other critical factors occurring within the same general time frame. Examples include severe weather events that impacted human health, political initiatives being established that was relevant. |
|  | - Top down support - The initiative would not have gone forward if it were not for the financial, technical or informational support of federal and / or provincial government. |
|  | - Respondents did not mention any drivers or enablers or could not identify any as of yet. |
| (a) Public Health Department legislation  (Ontario Provincial Public Health Standards) | - Adequate: Effective in providing clear and concise support and guidance and instruction on how to address climate change and public health issues. - Could Improve: Somewhat effective; however, incomplete in providing clear and concise support and guidance and instruction on how to address climate change and public health issues. - Not in a position to comment; does not know. |
| (b) Legislation for Non-public health departments | - Adequate: Effective in providing clear and concise support and guidance and instruction on how to address climate change and public health relevant issues. - Could Improve: Somewhat effective; however, incomplete in providing clear and concise support and guidance and instruction on how to address climate change and public health relevant issues. - Not in a position to comment |
| (c) Federal support | - Adequate: Effective in providing clear and concise support and guidance and instruction on how to address climate change and public health relevant issues. Support has been provided on addressing these issues and it has helped improve the level of preparedness. - Could Improve: Somewhat effective; however, incomplete in providing clear and concise support and guidance and instruction on how to address climate change and public health relevant issues. - Not in a position to comment; does not know. |
| **8. [*Posed to regional public health officials*] Would you please comment on the adequacy of intra-jurisdictional collaborations to guide your agency's ability to address the health impacts of climate change? Intra–jurisdictional refers to collaborations across divisions within your public health unit.** | |
| Collaborations within the health department could improve to increase the level of climate change preparedness: Communication and collaboration exist among staff members via pre-established networks and venues for meetings, but in terms of climate change, staff members could be interacting more often in order to maximize efforts on addressing climate change and public health related issues.  Strong collaborations; well established to address climate change and health issues: Communication and collaboration exist among staff members via pre-established networks and venues and are well established to incorporate climate change topics, that have either already happened, or are planned for the future.  Did not answer / Do not know: Lack of knowledge on the importance and / or adequacy of communication and collaboration across divisions within the public health unit in general and / or as it relates to addressing public health issues associated with climate change. | |
| **9. Would you please comment on the adequacy of inter-jurisdictional collaborations to guide your agency's ability to address the health impacts of climate change? Can you describe inter-collaborations in terms of partnerships between your [*public health department*] [*agency*] and (a) [*other*] regional and / or in municipal departments [*public health department*] (b) federal and / or provincial departments / ministries and (c) private or non-government organizations?** | |
| Collaborations could include written or oral communications or informational exchanges with other agencies.  Helpful / adequate: strong networks with agencies already, knowledge and information are gained to address issues related to climate change and public health.  Could improve: some interaction with other agencies, but information exchange is limited, the interactions could be stronger, occur more often, and could be better supported by other agencies given their knowledge and expertise.  No answer: not have enough confidence to know for sure, information is lacking, more research is needed or unsure of appropriate benchmark.  (a) Regional or municipal departments / other health units    (b) Non-government  (c) Provincial government  (d) Federal government | |
| **10. Would you please comment on the adequacy of existing programs and services, resources and staff to guide your agency’s ability to address health impacts of climate change? [*Posed to regional non-public health officials*] Do you have suggestions on how your agency must evolve to prepare for impacts of climate change in the future?** | |
| (a) Programs and services  Adequate: the programs and services in place are sufficient to be prepared for climate change  Could improve: programs and services are not where they could be, they could be stronger to address climate change.  Unsure if programs and services are sufficient: Do not know enough about how climate change will impact health to be able to determine if the programs and services are sufficient.  (b) Resources (financial, technical and informational tools)  Adequate: sufficient funding, technical and / or information tools are available or accessible to be able to address climate change.  Could improve: limited resources available and / or accessible to address climate change.  (c) Staff  Adequate: there are enough staff and knowledge and expertise among the staff are sufficient to address climate change.  Could improve: there is not enough staff or the knowledge and expertise among staff is insufficient to address climate change. | |
| **11. Would you please comment on the role of public health departments of Ontario in preparing for health impacts of climate change? Also, do you have suggestions on how [*your*] public health department[*s*] must evolve to prepare for health impacts of climate change in the future?** | |
| *Public Awareness and Outreach*: the primary role for public health units should be to engage, inform and educate the public and other municipal officials on topics relevant to public health climate change. This includes communicating the general and priority public health risks for the municipality. They are not necessarily the lead agency responsible for climate change in general; however, they play a significant role in communicating public health risks to the public.  *Integrate climate change into existing programs*: A primary role for public health units is to use existing resources to undertake the necessary research and surveillance initiatives in order to identify priority public health risks associated with climate change for the municipality and to identify the most effective responses to address those risks. They are not necessarily the lead agency responsible for climate change in general; however, should take the lead on proactive identification of priority public health risks and the most effective public health and / or collective community responses. Health units should take the leading role in the municipality to address climate change issues as they pertain to public health. They are responsible not only for identifying public health risks associated with climate change in the community, but also to establish appropriate responses and to share information with the rest of the community. They should allocate roles and responsibilities to other stakeholders in the municipality; arrange meetings and appropriate networks among municipal departments in order to address components associated with public health risks associated with climate change. They should be to understand how the weather and climate affect their programs and services and adjust them accordingly.  *Engage in partnerships and communication networks*: the primary role for public health units should be to regularly engage in communicating the needs of the public health unit with other municipal, provincial, federal and / or non-government entities in order to maximize knowledge and to identify resources for addressing potential health risks for the municipality. They are not necessarily the lead agency responsible for climate change in general; however, public health units should be proactively engaged in climate change discussions at various venues both at the municipal level and externally, with other municipalities, higher levels of government and non-governmental entities. This category was selected if there was a strong indication that public health adaptation is at an early stage of development and action by public health units should primarily be to stay tapped into climate change discussions, identify potential support resources and experienced or potential barriers to addressing climate change and public health risks.  Public health units can improve by:  *Stay tapped into climate change research:* Begin to or continue to be engaged on climate change adaptation science, and be aware of advancements on climate change and public health topics. Access and undertake more research on developing adequate responses. When necessary, lobby for local and provincial funding for climate change and public health work to be carried out via professional associations  *Identify needs and gaps* to mainstream climate change into existing programs: Increased integration of climate change into existing programs and services. Train public health unit employees on their roles in addressing climate change and public health risks.  *Improve partnerships*: Improved integration with municipal departments to communicate public health risks and potential responses.  *No change*: For one or more programs, public health units do not require major changes and should be prepared for future guidance and support from upper levels of government. | |
| **12. How do you presently see roles and responsibilities for climate change adaptation shared among levels of government and between government and non-governmental entities (e.g. NGOs, academia, private industry and the public)? What about in the future?** | |
| Federal government  Research and knowledge dissemination: a primary role for the federal government in general and / or specific departments or agencies is to conduct necessary research on topics to fill knowledge gaps on topics relevant to climate change and public health and to distribute the results to be used by provinces and municipal governments  Guidance documents and financial support: a primary role is to provide tangible and specific guidelines and / or financial support for the purposes of municipal adaptation to address public health challenges associated with climate change. For example, guidance and financial and / or informational support could be provided via best practice guides or funding for pilot projects to identify specific local climate change and health risks and to identify the most effective responses.  Inter-provincial / territorial and international partnerships: a primary role is to establish partnerships and / or agreements between governments of different provincial / territorial or national boundaries on topics relevant to public health and climate change. This role, for example, is critical considering those public health threats associated with climate change that cannot be addressed solely within jurisdictional boundaries of the municipality in question and / or when the source / cause of the public health threat originates within another Canadian jurisdiction or nation (examples of such public health threats include air quality, water quality and quantity and importation of foods from other countries).  National climate change strategy: a primary role for the federal government in general and / or specific departments or agencies is to establish a national strategy that includes adaptation to public health impacts of climate change. The federal government is responsible for providing collective leadership on preparing for climate change in Canada, which includes goals and objectives and strategies for the country to prevent climate change associated health threats.  Mitigation of greenhouse gases: a primary role is to ensure that Canada is actively working toward meeting national and international targets to reduce greenhouse gases. The Canadian federal government is responsible for fulfilling international obligations to mitigate greenhouse gases and fight global warming.  Other: respondents were unclear as to the best role for the federal government or chose not to answer for other unspecified reasons. | |
| Ontario Provincial Government  Partnerships with municipalities for build resilient communities: a primary role for the provincial government and / or specific ministries is to work closely with individual municipalities to assess public health risks of climate change and begin to address effective approaches to address those risks.  Identify common barriers and data needs: a primary role is to communicate with public health units and municipalities to identify challenges associated with adapting to public health impacts of climate change for the different localities, to summarize key barriers and data needs, disseminate findings in order to address how information and data gaps can be filled.  Regulations and policies: a primary role for the provincial government and / or specific ministries is to establish clear and tangible protocols, regulations and legislation on climate change and health.  Provincial adaptation strategy: a primary role is to provide leadership on addressing public health threats associated with climate change. This could be achieved via a provincial strategy with goals and objectives and implementation strategies to ensure that Ontario is taking the necessary steps to be adequately prepared for predicted health threats associated with climate change in Ontario. Included in this category are statements of general leadership on adaptation and / or on the need for improved horizontal communications, organization and role allocation for adaptation to climate change, specifically, for health relevant adaptations.  Public health adaptation inventory: Strong indication that a primary role is to establish a portal of best practices for addressing public health threats associated with climate change. The province is responsible for identifying innovative and evidenced based best practices for addressing public health threats associated with climate change and making them available to all public health units and interested municipalities in the province and where interested, externally (e.g. other provinces / territories and internationally).  Proactive engagement and financial support for adaptation: a primary role for the provincial government is to fund regional and local efforts to address climate change and health risks.  Other: respondents were unclear as to the best role for the provincial government or chose not to answer for other unspecified reasons. | |
| Regional or Municipal government  Respondents commented on the importance of integrative and collaborative efforts, designating leaders and mainstreaming adaptation into existing services and programs.  Integrative and collaborative planning: a primary role for the municipal government as a whole is to establish venues and mechanisms for regular communication, collaboration and brainstorming in order to identify priority public health risks for the community and effective and collective ways to address those risks.  Designate a leader and identify stakeholder roles: a primary role for the municipal government as a whole is to identify the municipal department and individual(s) who are well positioned to adopt a leadership role in managing and organizing meetings, drafting plans and strategies and leading discussions at communication venues. With respect to identifying priority public health threats of climate change and the effective responses, the appropriate stakeholders should be identified and their roles and responsibilities clearly defined. Key stakeholders may be housed mainly within the public health department and / or across other municipal departments.  Mainstream climate change into existing programs and services: a primary role for the municipal government as a whole is to ensure that the adequate steps to minimize public health threats associated with climate change are carried out. The municipal government departments are responsible for integrating these actions into existing programs services and / or creating new programs to ensure that actions are implemented. | |
| Non-government  Respondents commented on the role non-governmental groups in providing research and program support and for direct public outreach.  Research and program support: a primary role for non-governmental groups is to conduct the necessary research to fill climate change adaptation and public health relevant knowledge and data gaps and to provide the informational support and guidance to improve on existing or newly established programs and services to minimize health risks associated with climate change.  Public outreach: a primary role of non-governmental groups is to provide direct services for the public, particularly vulnerable segments of the population who may have limited resources and support during episodes of extreme weather and other health threats associated with a changing climate. | |
| General Public  Respondents commented on the importance of the public in staying informed, being environmental stewards and changing behaviour.  Stay informed: a primary role of the public is to remain aware of climate change and risks to public health through government outreach and messaging on the topic and / or independent learning and research. Also, a main role of the public is to assist in efforts to lobby for political change; specifically, for public health and climate change policies for the province and / or municipality and / or specific community.  Environmental stewardship and behaviour: a primary role of public citizens is to change personal behaviours and practices in order to be environmental stewards and to be better prepared for potential public health threats associated with climate change in one’s own community. Individuals have a role to educate and encourage others to do the same. | |
| **13. Would you please suggest three things that need to change in order for your [public health department] [agency] to be better prepared to address the public health challenges of climate change? Please provide an explanation for each.** | |
| Public Health Department and Regional Level Action  Adjust programs and services when necessary: the public health and other regional agencies should continue to or begin to actively seek out resources that will help inform improvements or alterations to existing programs and / or to create new programs or services to be better prepared to minimize health risks associated with climate change.  Maintain existing levels of effort and remain engaged and tapped into research and guidance: maintain existing efforts to address climate change are high. It is important to remain engaged on the topic and to stay tapped into research findings, best practices and other useful information to be best prepared.  Community awareness and knowledge exchange: This theme included a variety of responses that are summarized below.   - Public awareness: Strong indication that public health officials and / or others in the community need to do a better job at increasing awareness on the public health risks associated with climate change to public citizens within their jurisdiction. Statements were made that in general, the public are not making the link between climate change and health and is likely due to a lack of understanding of climate change and adaptation and / or associate climate change with other non-health factors like greenhouse gas mitigation and melting polar ice caps, declines in polar bear populations. In terms of public health, individuals tend to consider health impacts to populations in developing countries, where individuals are far more significantly impacted by extreme weather events much more so than people living in Ontario. Public health units are responsible for engaging the public on the topic. This could be achieved by making climate change personal to individuals, presenting meaningful costs of inaction that will affect the individuals health, that of their family and / or their community. Public health units should educate the public on the challenges of addressing climate change, for example, because of the uncertainty in predicting future weather patterns at their local level, and promote widespread messaging on the importance of proactive research and necessary steps in order to address these challenges. Public awareness and education was stated as critical, for example, in order to push for policy changes at senior levels of government. Also included here are increased communications with civil society groups to inform them of how they can contribute to minimizing public health threats of climate change in the community. - Engage municipal council and municipal government departments: Strong indication climate change b more of a priority at the regional priority. Public health officials and / or others in the community need to be communicating with their municipal council and senior management across other municipal departments (e.g. water and utilities, planning, infrastructure and energy, parks and recreation, transportation etc.) about climate change and how it will impact the health of the public in their community. Public health units need to work toward making convincing cases to the municipal government(s) senior staff to be engaged on the topic, think about what it means to their own standards and operations and to present the topic from a public health lens. Public health units should work toward multidisciplinary discussions on how to address public health threats associated with climate change and that are predicted for their community. - Capitalize on information sharing venues: efforts should continue to be made to share information at pre-existing and / or new venues for discussion on climate change and public health topics. Such venues exist in the form of webinars, conferences, committee meetings and / or other venues for learning and knowledge exchange.   *Other suggestions – Research and Support Tools, Leadership*   - Surveillance and Monitoring: effort needs to be put toward identifying necessary environmental exposure data that is needed before further action is taken in terms of setting up programs and establishing policies. Respondents stated that a key limitation is not having the necessary data to move forward on planning for the future. Health data exists in many forms and in many places; however, it is not always easily accessible or readily available or in a form useable for the purposes of addressing cc. Environmental exposure data and / or linking public health impacts with climate related environmental factors are currently limited for effective policy and programming. This has been stated especially for vector borne, water borne and food borne diseases and air quality. - Guidance and Protocols: effort needs to be made to provide tangible guidelines and protocols to address climate change and public health issues. Public health departments need guidance on how to identify and prioritize climate change related health risks and how to undertake proper risk / impact / vulnerability / adaptive capacity assessments. They need to know what data should be collected, how to collect, where to obtain it and what resources they should be using to obtain it. Where they are unable to obtain the data, effort should be made to collect it and make it available to them as much as possible. Many respondents noted that both the province and the federal government departments should continue efforts in this regard. - Inventory of best practices and research output: Some public health units are actively engaged in addressing climate change (e.g. are involved in municipal level climate change adaptation strategies, have undertaken vulnerability assessments, and some are implementing specific actions to address particular climate change and public health threats). Effort should by provincial health ministry’s to identify how different public health units are preparing for climate change in their communities, highlight best practices and establish a portal for housing the information in order to make it available to all health units in the province. Research to identify informational and data needs: effort needs to be made by the provincial health ministries to research public health and climate change impacts for different regions in Ontario. Further, the provincial health ministries should identify the barriers to preparing for climate change across public health units in Ontario. Many respondents noted that both the province and the federal government departments should continue efforts in this regard. - Funding: effort should be made toward allocating dedicated funding to climate change activities for public health units to be able to take steps to address the climate change related priority health risks for their area. - Effort should continue to be made toward improving modelling and forecasting of future climate at the local level. Efforts should be made to make the available readily available to public health units and other departments in the municipality in order for policy planning and programming for the future. - Effort should be made toward establishing a national climate change adaptation strategy that includes goals of addressing public health threats for the different regions of Canada   No response: the participant provided a very general answer that was too difficult to categorize, for example: “we need more funding and resources” or they did not provide an answer. These responses were more common during group interviews, when there was only time for one person to respond. | |
