# Supplementary material for: Adaptation to climate change in the Ontario public health sector
Source: BMC Public Health. 2012 Jun 19;12:452. doi: 10.1186/1471-2458-12-452 (PMC3418204; doi:10.1186/1471-2458-12-452)
Supplement: Additional file 7 — Additional representative quotes. [file 1471-2458-12-452-S7.docx]

7. Additional representative quotes

| **Key public health unit adaptations include mainstreaming climate change, promoting awareness and interdisciplinary participation on addressing climate related health risks**  *There is quite a bit of disparity in terms of preparedness for cc impacts at the municipal level across Canada; very few have actually done any type of scan or assessment of their vulnerability. Toronto and Montreal are exceptions. One area where there has been some progress is the awareness of public health departments that climate change is a real issue for them and they need to do something about it. That's the first big step. ..If assessments indicate that risks will increase or have increased, public health units will need to martial their resources and review organizational capacity to change their programs to protect people (Federal public health official)*  *In terms of adaptation we must ask ourselves: how are we going to react in the event that we are going to see more storm events, more diseases, like vector-borne diseases? This is needed rather than waiting for these things to happen. Let’s get some processes and plans in place so we are ahead of the way (Municipal public health official)*  *It is our job to collect information and make it available. It’s up to us to determine what we need to do to have adequate programs .A lot of the work we do [in environmental health] is preventative, in terms of health promotion and advocacy, we want to avoid emergency situations from happening, our heat program is very preventative in nature. We put the word out there before health is negatively affected (Municipal public health official)*  *We are a community leader in adaptation not mitigation; however, we are a proponent of mitigation to minimize long-term impact. In terms of adaptation we must ask ourselves: how are we going to react in the event that we are going to see more storm events, more diseases, like vector-borne diseases? This is needed rather than waiting for these things to happen. Let’s get some processes and plans in place so we are ahead of the way (Municipal public health official)*  *One of the things local public health units can do is to identify areas of critical weakness and work hard to have those addressed. Use climate change as a motivating argument where appropriate and be proactive in ensuring that those changes get made (Provincial public health official)*  *The bulk of their role is adaptation. Negative things are going to happen as a result of climate change. So there needs to be adaptation in the public health region. Public health units are an important -partner in monitor trends, identifying risks across the full range of potential climate related risks and deciding on best ways to improve the situation. They have to be a partner in looking at those areas because they have regional knowledge. I think they have a very good foundation, already have programmatic areas in place; they just need to add what's out there for knowledge that already exists on cc to what they already have in place (Federal public health official)*  *There is quite a bit of disparity in terms of preparedness for cc impacts at the municipal level across Canada; very few have actually done any type of scan or assessment of their vulnerability. One area where there has been some progress is the awareness of public health departments that climate change is a real issue for them and they need to do something about it. That's the first big step. There is a lot of work that needs to be done…They need to identify if climate change will be a risk for the health of people in their communities and get information on these risks, many of which are trying to do that right now, and then figure out what to do about it. If assessments indicate that risks will increase or have increased, public health units will need to martial their resources and review organizational capacity to change their programs to protect people. This involves knowledge generation and program development function. They also have to advocate for the reduction of GHGs (Federal health official)*  *We are supposed to anticipate health impacts from any potential health hazard and respond to it. Preparedness in terms of cc for us means doing surveillance and putting plans in place in the event that something happens (Municipal public health official)* | **Public health officials are concerned about climate change impacting human health**  *I think air quality is probably is one of the big ones. We already know it’s big. From our shop produced reports, poor air quality contributes to 6000 hospitalizations and 1700 deaths / yr in the city. There is good evidence to support this (Municipal public health official)*  *We will see more hot days; it is certainly something that will need to be addressed. There are definitely post-flooding health issues. Mould in basements is one (Municipal public health official)*  *Infrastructure is not up to date. We have a combined sewer system, so in a flooding situation, untreated water goes straight to the river (Municipal public health official)*  *I am more concerned with private water systems, like wells. Water quality is very important. Warm weather combined with animal and human waste in water and contaminants in rain affects water quality in private wells (Municipal public health official)*  *Drought and water quantity and quality is a concern for us. In 2007, we had a supply issue to the point where we didn’t have enough water at the fire department for all municipalities (Municipal planner)*  *There are some [climate change risks] we know a lot more about than others, so heat and air are the two where we have the most information, so no matter what, I would say they’ll certainly appear and appear to be the two most critical factors. I think air quality is probably is one of the big ones. We already know it’s big. From our shop produced reports, poor air quality contributes to 6000 hospitalizations and 1700 deaths / yr in the city. There is good evidence to support this (Municipal public health official)*  *The primary impact is likely to be increased extreme weather (Municipal public health official)*  *We will see more hot days; it is certainly something that will need to be addressed. There are definitely post-flooding health issues. Mould in basements is one (Municipal public health official)*  *Extreme heat and cold are among our top three concerns based on our risk assessment (Municipal public health official)*  *Infrastructure is not up to date. We have a combined sewer system, so in a flooding situation, untreated water goes straight to the river (Municipal public health official)*  *Water quality is a concern. This is a recreational water use health concern more so than a drinking water (Municipal public health official)*  *With climate change we may see more extreme weather, more flooding. I am more concerned with private water systems, like wells. Water quality is very important. Warm weather combined with animal and human waste in water and contaminants in rain affects water quality in private wells. This affects the safety for people in rural areas. We have had contamination in wells that were weather related (Municipal public health official)*  *We already have air quality issues due to our proximity to major highways and being highly industrial; climate change will exacerbate that With more heat we will see more respiratory disease via smog formation; People will be outside more if there are more warm days, so more outdoor activity will likely affect respiratory issues (Municipal public health official)*  *We are reliant on ground-water in a number of communities. Drought and water quantity and quality is a concern for us. In 2007, we had a supply issue to the point where we didn’t have enough water at the fire department for all municipalities (Municipal planner)*  *There have been many beach closures in recent years due to high temperatures and propagation of E. Coli. If we have longer warm periods and more hot days, this problem will worsen (Environment official)*  *Lower water-levels in our lakes associated with climate change will lead to more boating accidents in the summer time (Municipal public health official)*  *We experienced the impact of the wood smoke in Ottawa from the Quebec fires this year – we realized that we are vulnerable to that kind of pollution (Municipal public health official)*  *Most food is imported to Toronto. My main concern is the source of the food and inadequate screening at the federal level and the levels that follow from there. High risk of food contamination in other countries where climate change has had impacts; for example on fisheries or agricultural goods, we will see those issues translated here*.....*Campylobacter on livestock and poultry increases with environmental stresses, this includes heat and drought. So climate change may affect the food that is produced here in Canada that we eat locally. Also, the number of food recalls will go up with climate change (Municipal public health official)*  *We see food contamination and illness spikes in the summer. This is attributed to heat. Also, During the summer, people are outside barbequing more, there are more outdoor and catered events, longer summers mean more of this type of activity which will likely influence food borne illness incidences (Municipal public health official)*  *We are seeing more food-borne illnesses in Canada, for example, Listeriosis, so there is a risk for us locally (Municipal public health official)*  *We have Lyme disease here; we have the ticks that carry Lyme disease. A region south of us is endemic with deer ticks, so the disease could find its way here, working its way up via birds and animals. We are seeing some mosquitoes as part of our West Nile Virus Program that can transmit malaria and dengue. The possibility of these viruses turning up here is something we think about (Municipal public health official)*  *Vector borne diseases [VBD] are a risk. The challenge [with VBD] is that it's a complex science; science tells us that diseases may end up establishing themselves. It’s happening in States (Florida I think and others). Dengue is not prevalent in the US, but they have the climate for it. The conditions are there to support it. If the vector is there, we just need the pathogen to come and then it could very well spread (Municipal public health official)*  *I am biased on this one. We have mosquitoes for malaria, so we need to be aware and recognize the potential for malaria coming here again - used to be here in Ontario. With the globalization we are seeing, it's hard to ID weather globalization or cc - but both likely. Combining these different aspects together is what's really interesting and challenging. For Lyme - we don't have established populations of deer ticks, but we do monitor it (Municipal public health official)*  *We are certainly seeing changes. For example, Eastern Equine Encephalitis was found south of great lakes. It has a significant fatality rate associated over last two summers, we've seen it appear in Ontario for first time. So, is it coming? Well, we are tracking it closely. If it starts to come up, it could have considerable health impacts. Lyme disease happened first around Kingston, Frontenac and recently in Niagara. We have right conditions in Peel, so it’s a matter of time, we are doing surveillance for it (Municipal public health official)* |
| --- | --- |
| **Climate change is not addressed as a stand-alone issue**  *It is low in terms of work done, but in our minds it’s higher; for example, in emergency planning for health hazards, it’s medium to high for us (Municipal public health official)*  *Climate change has crept into our programs; we recognize its importance. We try to integrate it into our health promotion pieces on extreme heat and air quality. Our heat plan started in 2010. As we plan and work toward addressing heat, urban sprawl, minimizing single occupancy vehicles and implementation of our heat and air quality plans, more and more cc is being integrated into it. We acknowledge climate change in our outreach material – climate change are the first two words in our heat pamphlets (Municipal public health official)*  *The heat program originally started in 1999 in response to elderly and vulnerable groups voicing concerns about the health issues associated with heat, so climate change wasn’t a motivating factor back then. Over time, cc became integrated. CC spans many issues across the city and was pulled into the heat program because cc affects heat, so it affects health (Municipal public health official)*  *We don’t have a program called ‘climate change’, but it underlies a lot of the programmatic activity we’re involved in. We don’t need a climate change division or climate change person; we will just integrate climate change into existing activities*. *Everything we do is evidenced based. We have epidemiologists looking at burden of illness patterns that are linked to climate change. We are forward thinking. (Municipal public health official)*  *It is low in terms of work done, but in our minds it’s higher; for example, in emergency planning for health hazards, it’s medium to high for us (Municipal public health official)*  *Weather is a priority, not climate change. Climate change is under health hazard section of our standards, as an inspector, you deal with day to day hazards, not long-term issues, so what do we do about it? Well, we don’t know what to do. We are part of the whole municipal emergency control system and we would certainly be looking at climate change health related issues in any kind of an emergency; so a power outage, we talk about food safety, because peoples fridges and freezers are down; water quality if someone is on a private system, that’s education role for us (Municipal public health official)*  *We have [name of research and policy person] who does our climate change work mainly, it is one of our areas that we have been working on, it is part of what we do, but not a big priority. It has been really integrated in to our heat response program (Municipal public health official)*  *In the developing world it [climate change] is more of a [public health] issue. We have a resilient community, we aren’t on the coast, and there is no risk of typhoons or hurricanes, despite some increases in some unstable weather patterns. To a certain extent, we have to deal with immediate problems. Our emphasis is on immediate and pressing health risks and ongoing projects.....* *cc will make existing issues worse, but we already have programs in place for these, for example heat and sun safety (Municipal public health official)*  *We are mandated to inspect food premises by the province. So climate change is relevant and something to consider (Municipal public health official)*  *(1) The HARS project was established on the premise of cc; but within the framework of emergency management plans and policies and public health, it wasn’t the real driving factor in our heads. It was just one component (2) Climate change was the initial rationale for the extreme heat response program, but was not exclusively driving it. We needed to address health threats associated with extreme heat in the City ((1) Municipal emergency manager, (2) (Municipal public health official))*  *Climate change has crept into our programs; we recognize its importance. We try to integrate it into our health promotion pieces on extreme heat and air quality. Our heat plan started in 2010. As we plan and work toward addressing heat, urban sprawl, minimizing single occupancy vehicles and implementation of our heat and air quality plans, more and more cc is being integrated into it. We acknowledge climate change in our outreach material – climate change are the first two words in our heat pamphlets (Municipal public health official)*  *The heat program originally started in 1999 in response to elderly and vulnerable groups voicing concerns about the health issues associated with heat, so climate change wasn’t a motivating factor back then. Over time, cc became integrated. CC spans many issues across the city and was pulled into the heat program because cc affects heat, so it affects health (Municipal public health official)*  *Climate change is an aspect of our vector borne disease programming and emerging zoonotics - it's on our radar and we consider it, we are looking into it. We acknowledge that through changing weather patterns there is a direct relationship between diseases and climate change. We know that accumulated degree days speeds up the hatching process and can generate more mosquitoes with hotter weather. We monitor weather for this purpose. We are mandated by the Ontario Public Health Standards, but over the years our program evolved based on what we've learned, including what has come from surveillance activities. We know the potential for Lyme disease is there. We have to consider effects of migratory birds bringing the disease here (Municipal public health official)*  *Our vector borne disease programming is not explicitly climate change, but we include it in our discussions. In our West Nile Virus Plan, we include its relevance in the report (Municipal public health official)*  *We don’t have a program called ‘climate change’, but it underlies a lot of the programmatic activity we’re involved in. We don’t need a climate change division or climate change person; we will just integrate climate change into existing activities*. *Everything we do is evidenced based. We have epidemiologists looking at burden of illness patterns that are linked to climate change. We are forward thinking (Municipal public health official)*  *The healthy communities’ initiative promotes walk-ability in neighbourhoods and greening initiatives. This is relevant to mitigation and adaptation, but it’s not really couched as that (Municipal public health official)* | **Key adaptation enablers include political will, inter-agency coordination and local leaders**  *Communicating with the federal government is important in our work. For example, Health Canada provides us with updates and information on their heat alert and response project. They update us on research, and give us information on what they are developing. Environment Canada give us climate data and information on our heat program - so we can issue humidex alerts (Municipal public health official)*  *Our previous council passed a resolution saying climate change was one of the most urgent issues of the century. This allowed for the adaptation planning to start (Municipal planner)*  *We work with all divisions in the city on climate change issues, including health. Through our climate change committee participation, there are representatives from key divisions in the city. From that perspective, it has been a good opportunity to hear from other divisions and have the ability to collaborate (Municipal public health official)*  *The Ontario Public Health Association (OPHA) is important because we have the built environment working group. There is a lot of potential there for sharing and learning. Highly resourced health units are part of those. They have the capacity for trial and error and develop more expertise in house and can communicate findings and ideas at the OPHA. Little health units like ours can benefit from the information that comes out of there (Municipal public health official)*  *We work with all divisions in the city on climate change issues, including health. Through our climate change committee participation, there are representatives from key divisions in the city. From that perspective, it has been a good opportunity to hear from other divisions and have the ability to collaborate (Municipal public health official)*  *Building partnerships with the appropriate departments of any level of government, NGOs and academia mostly to share information and ensure a common understanding of available data is really important. Communities will then be able to design adaptation strategies appropriate to their own circumstances (Municipal public health official)*  *We have a responsibility to be positive, because if we aren't, what hope is there. We have to work toward understanding and changing and be intelligent about it. We can turn things around - it's hard to do but we have to. We just have to accept that it's not a simple fix. Recognizing existing capacity and resiliency level is relatively very good to other countries and using that to empower leadership and moving forward, not an excuse to do nothing (Municipal environment official)*  *Climate change and health was on the radar prior to the Heat Alert and Response System (HARS) project, but HARS helped us with the relevant planning at the local level. In terms of vulnerable groups, we received lots of guidance from Health Canada.* |
| **Adaptation constraints include inadequate resources, a perceived lack of urgency and communication barriers**  *[flood risk] Plans are based on weather events that have occurred in the past - if those predictions are wrong, then they need to be revised to account for more rain or whatever the case may be (Municipal environment official)*  *We are supposed to anticipate health impacts from any potential health hazard and respond to it. Preparedness in terms of cc for us means doing surveillance and putting plans in place in the event that something happens… We need more environmental exposure data to guide our actions. One of our hardest challenges deals with linking environment with health outcomes. Our work needs to be evidenced based (Municipal public health official)*  *We don’t have the resources to do trial and error of what works and what doesn’t. What we need is a one stop shop and easy at our fingertips best practices for making plans for these health hazard emergencies (Municipal public health official)*  *We know what we need to do, but we need the funding to do the work*. *We are inadequate to deal with some of the programs we want to initiate because of budget constraints; decreased support from higher government for funding (Municipal official)*  *On hot days cool yourself by using air conditioning; however, if it’s hot, it could also be a smog day. So we tell them to use your AC for heat, but not to use your AC for smog. These programs are in different areas of the health department, so we are inadvertently sending out conflicting messages. This is a significant challenge for health units (Municipal public health official)*  *Our programs and services may be well positioned, could be relevant, but they are not carried out through cc lens. For example, in our extreme weather program - cc links aren’t here - so there is definite work to be done. Programs don't have enough recognition and integration of the concept of climate change (Municipal public health official)*  *[flood risk] Plans are based on weather events that have occurred in the past - if those predictions are wrong, then they need to be revised to account for more rain or whatever the case may be (Municipal environment official)*  *The work [cc impact assessment]* *will involve a reasonable amount of educated guessing on my part because cc models typically are at the hemispheric or global level it's hard to pin down precise impacts regionally and locally in particular. There are all kinds of questions that we don’t have the answers to (Municipal public health official)*  *We are starting to develop a surveillance plan for all of our programs, but it’s really hard to 100% associate respiratory outcome with actual impact right because it could be something totally different. It could be air quality or heat. That is one of our hardest things. Even the water borne diseases, we may know how many people have E. coli infections, but don’t know what percentage of people food or water borne, there is a big gap in Ontario with information with actual exposure surveillance, meaning, we know how many have e coli, but how many of those, are they able to identify 100 % close link to consumption of waterWe need to be confident with our plans. We need more environmental exposure data to guide our actions. One of our hardest challenges deals with linking environment with health outcomes. Our work needs to be evidenced based (Municipal public health official)*  *Our number one problem is lack of human and financial resources to create adequate and sustainable programs on climate change. We don’t have the resources to do trial and error of what works and what doesn’t. What we need is a one stop shop and easy at our fingertips best practices for making plans for these health hazard emergencies (Municipal public health official)*  *We know what we need to do, but we need the funding to do the work*. *We are inadequate to deal with some of the programs we want to initiative because of budget constraints; decreased support from higher government for funding. If we don't get enough provincial funding and at the same time the mayor is not giving money for the initiatives; the money has to come from somewhere. At this time, I don't think it's adequate* *(Municipal planner)*  *So for climate change, health risks that we don’t have evidence for yet, the funding won’t be there; we re-allocate resources, based on surveillance - on positive human cases in our area but with several positive mosquitoes, we didn’t get any cases here. If nobody is falling ill with the disease, then there is no point in putting resources into programs when they could be targeted to higher and more immediate health risk needs (Municipal public health official)*  *I have a feeling that we aren't necessarily in total control on climate change; we can bring issues up of course, but still I think we are just so focused on our general mandated responsibilities, we are not really going beyond that in any way to be effectively addressing climate change (Municipal public health official)*  *We* *have to identify ways to establish more long-term funding and resources to ensure that we have permanent modeling equipment on those beaches. It comes down to finding avenues to fund them. This is an ongoing challenge (Public health official, Niagara)*  *We have an intern working on the Adaptation Consortium but the contract is only good for a number of months. Work needs to be done continually to keep it going. We don't have a dedicated ability to do that day in and day out month to month (Regional conservation official)*  *I wish it was more of a priority. It’s definitely on our radar; however, there are a lot of competing issues. In my personal opinion, we need to be doing more (Municipal public health official)*  *In terms of creating the response plans for each health hazard, we need more information on what the most effective messages are; for example, communicating with vulnerable groups prior to the emergency and during and right after (Municipal public health official)*  *I am unsure how the political arena will influence sustained work in future. There will be competing financial priorities and agenda items. The City produced the funds to do the risk assessment-a necessary but very expensive project (Municipal environment official)*  *It is low in terms of work done, but in our minds it’s higher. It is a lack of human resources that limits specific work on the topic (Municipal public health official)*  *The local food programs will likely take 10-15 years to see changes and results. It is very hard to know if this will keep momentum with changing governments (Municipal public health official)*  *On hot days cool yourself by using air conditioning; however, if it’s hot, it could also be a smog day. So we tell them to use your AC for heat, but not to use your AC for smog. These programs are in different areas of the health department, so we are inadvertently sending out conflicting messages. This is a significant challenge for health units (Municipal public health official)*  *Resources and funding not a la carte; but we find ways to get them. The real problem with respect to cc is not existing programs etc. but uncertainty about future weather (Municipal public health official)*  *It has been a high priority at the city level; the issue has been raised in various city initiatives, but this was under our last government. The future priority will depend on the new council priorities (Municipal environment official)*  *(1) We are at the beginning stages of communicating with community partners on the climate change adaptation topic. This includes identifying risks and brainstorming best responses. Although it is starting to happen, we still need a lot of work. Again, what are stopping us are the resources*.  *(2) We don’t have enough coordination internally and externally. We need a one stop shop for climate change to tie our programs and services on cc together. We are too disconnected now ((1) Municipal planner (2) Municipal public health official)*  *I think we can do more; we need a cc strategy for public health. Now we have a whole bunch of programs that are just sort of out there, dealing with a particular issue, other than that, there is nothing strategic. There should be a cc strategy - it's hard to get everything organized and move everything forward until you have something to point to saying and reminding - this is what we are doing and why we are doing it. In terms of integrating the need for cc across branches and health topics in PH - we need a cc adaptation strategy that will be relevant for all topics (Municipal public health official)*  *Our programs and services may be well positioned, could be relevant, but they are not carried out through cc lens. For example, in our extreme weather program - cc links aren’t here - so there is definite work to be done. Programs don't have enough recognition and integration of the concept of climate change (Municipal public health official)*  *We have a lack of trust in knowledge on climate change risks to be sure that our programs are adequate to address climate change (Municipal public health official)*  *There needs to be more research to determine this. Any qualitative information would be valuable (Municipal public health official)* | **More support from the province and federal government is needed for local adaptation**  *It is a big step forward for the standards to have cc in there specifically. In 2008 the concept was still emerging and evolving... It is my hope that the next iteration will give more guidance on what the provinces expectations really are... Public Health units would benefit if their role was more clearly articulated (Municipal public health official)*  *Our climate change action plan was absolutely bottom up. We recognized internally that cc is something that will impact the region and should be incorporated and it wasn’t something the feds or the province mandated. We would see much more progress if we had more higher level support, like if it was mandated (Municipal planner)*  *Ministry of HLTC not looking closely at it. Even when we bring it to their attention, the ministry ID's the expert panel, not sure they take it seriously yet (Municipal public health official)*  *Unless they get serious about it, no one else will. They need to put resources into it. Funding needs to go with instruction from the province; province could be providing dedicated funding to the new agency to do the necessary research; in order to identify the cc related risks and act on them, to do this work, we need the funding for it. We know how to do assessments, we need to have more information about cc and health and then go out and do what needs to be done, but can't do it without the resources or the sense of urgency (Municipal public health official)*  *Something that we don't have that we do need is a federal adaptation strategy because what that would do is provide us with guidance on how we engage with provinces and municipalities; this is something we are hoping is developed, we don't have that yet (Federal public health official)*  *It is a big step forward for the standards to have cc in there specifically. In 2008 the concept was still emerging and evolving. We didn't have all the answers at the time, good place to start. We are at the stage of trying to identify what we need to be concerned about, and how to start to integrate that into our long-term planning program. It is my hope that the next iteration will give more guidance on what the provinces expectations really are. So it's a good start, but there is room for more. PH units would benefit if their role was more clearly articulated (Municipal public health official)*  *Outside of emergency planning there needs to be more about cc for sure. The climate change piece with respect to emergency response is very relative. But looking at other adaptations and how to do that, and how to plan for that for your community, we don’t' have enough expertise, knowledge or experience with that*. *Public health units could have a much bigger role than just "communication" and the extent of the actions should be embedded in the standards* *(Municipal public health official)*  *In terms of cc, could be doing more especially for those health units with very little resources, if cc was more identified in OPHSs it would be easier (Municipal public health official)*  *Our climate change action plan was absolutely bottom up. We recognized internally that cc is something that will impact the region and should be incorporated and it wasn’t something the feds or the province mandated. We would see much more progress if we had more higher level support, like if it was mandated (Municipal planner)*  *Ministry of HLTC not looking closely at it. Even when we bring it to their attention, the ministry ID's the expert panel, not sure they take it seriously yet (Municipal public health official)*  *Unless they get serious about it, no one else will. They need to put resources into it. Funding needs to go with instruction from the province; province could be providing dedicated funding to the new agency to do the necessary research; in order to identify the cc related risks and act on them, to do this work, we need the funding for it. We know how to do assessments, we need to have more information about cc and health and then go out and do what needs to be done, but can't do it without the resources or the sense of urgency (Municipal public health official)*  *There is so much data that we need form the different levels of government.* *We need things from them that we can't afford (e.g. weather data). We need the feds to do their global and regional cc models. Models will help municipalities better understand the potential future changes in their area. Impacts are best addressed at the local level (Municipal planner)*  *There is the technology out there but not the funds. Ontario doesn’t have its own cc organization like Quebec and British Columbia. The Expert Panel in Ontario doesn’t do modelling or research; they indicated their opinions on suggested directions. They say that more climate science is needed (Municipal environment official)*  *The new Agency of Health Protection and Promotion in Ontario will be important. What would help this agency, are positions and policies that may come down from the federal level, because the OAHPP can't do it on their own - this is a big big topic - all need to work together on this issue...for more dedicating resourcing on dedicated cc work in the localities - more research from agency and feds to help change policy - derives mandates and that's where you get funding to hire people to get that proactive in it. Now, we don't know that much - it's proactive and working with inner agency folks, working together, brainstorming, we are early on. We need the research to push the agenda - research should look at models that work and make a difference. This work will begin to inform best practices (Municipal public health official)*  *We know what the thoughts on the feds minds are [NR Can], there used to be more support or resourcing or commitment [on climate change issues], which seems to have gone away. We are limited with resources, we are busy with day to day work, and do not have sustained resources to do the climate change adaptation work. We need more financial support (Regional conservation official)*  *Something that we don't have that we do need is a federal adaptation strategy because what that would do is provide us with guidance on how we engage with provinces and municipalities; this is something we are hoping is developed, we don't have that yet (Federal public health official)* |
